# High levels of Daxx due to low cellular levels of HSP25 in murine cancer cells result in inefficient adenovirus replication

**DOI:** 10.1038/s12276-019-0321-4

**Published:** 2019-10-15

**Authors:** Zhezhu Han, Yeonsoo Joo, Jihyun Lee, Suwan Ko, Rong Xu, Geun-Hyeok Oh, Soojin Choi, Jeong A. Hong, Hye Jin Choi, Jae J. Song

**Affiliations:** 1 Institute for Cancer Research, Yonsei University College of Medicine, Seoul, Korea; 2 Department of Oncology, Affiliated Hospital of Yanbian University, Yanji, Jilin Province PR China; 3 Severance Biomedical Science Institute, Yonsei University College of Medicine, Seoul, Korea; 4 Department of Internal Medicine, Yonsei University College of Medicine, Seoul, Korea

**Keywords:** Biochemistry, Medical research

## Abstract

When the adenoviral protein E1B55K binds death domain-associated protein (Daxx), the proteasome-dependent degradation of Daxx is initiated, and adenoviral replication is effectively maintained. Here, we show that the cellular levels of Daxx differ between human and mouse cancer cell lines. Specifically, we observed higher cellular Daxx levels and the diminished replication of oncolytic adenovirus in mouse cancer cell lines, suggesting that cellular Daxx levels limit the replication of oncolytic adenoviruses that lack E1B55K in murine cells. Indeed, the replication of oncolytic adenoviruses that lack E1B55K was significantly increased following infection with oncolytic adenovirus expressing Daxx-specific shRNA. Cellular Daxx levels were decreased in mouse cells expressing heat shock protein 25 (HSP25; homolog of human HSP27) following heat shock or stable transfection with HSP25-bearing plasmids. Furthermore, Daxx expression in murine cell lines was primarily regulated at the transcriptional level via HSP25-mediated inhibition of the nuclear translocation of the signal transducer and activator of transcription 3 (stat3) protein, which typically upregulates Daxx transcription. Conversely, human HSP27 enhanced stat3 activity to increase Daxx transcription. Interestingly, human Daxx, but not mouse Daxx, was degraded as normal by ubiquitin-dependent lysosomal degradation; however, HSP27 downregulation induced the ubiquitin-independent proteasomal degradation of Daxx.

## Cancer: Using viruses as therapy

Cancer therapies that use a virus to kill tumor cells may get a boost by suppressing a common, ubiquitously expressed protein called Daxx. The relatively new field of virotherapy uses engineered adenoviruses, which usually cause fevers, coughs, or sore throats, to attack tumor cells, enabling treatment of advanced stage cancers, or those that have spread through the body. However, the immune system can attack the therapeutic virus, preventing it from replicating and reducing its effectiveness. Hye Jin Choi and Jae Song at Yonsei University, Seoul, South Korea, and coworkers have been investigating ways to maximize replication of the therapeutic virus. They found that suppressing Daxx improved viral replication; further testing showed that suppressing Daxx acted via different mechanisms in mouse and human cancer cells. These results will help develop more effective virus-based cancer therapies.

## Introduction

Daxx is a multifunctional, ubiquitously expressed protein implicated in various critical cellular functions such as transcriptional regulation, apoptosis, carcinogenesis, and antiviral protection^1^. Originally, Daxx was thought to function as a repressor or activator of gene transcription^2^; however, Daxx has also been found to influence transcriptional activity by interacting directly with other nuclear transcriptional factors including E26 transformation-specific sequence 1 (ETS1), paired box gene 3 (PAX3), paired box gene 5 (PAX5), androgen receptor, p53 family proteins, SMAD family member 4 (SMAD4), and glucocorticoid receptor to act as a transcriptional corepressor^3^. Daxx is also a CD95-binding protein that activates c-Jun NH2-terminal kinase (JNK) via directly binding to apoptotic signal-regulating kinase 1 (ASK1)^4,5^. Studies have also implicated Daxx in viral replication^6–11^. We previously observed that silencing of the Daxx gene increased oncolytic adenoviral replication^11^, which is consistent with the fact that effective adenoviral replication is maintained only when the adenoviral protein E1B55K binds to Daxx and triggers its proteasome-dependent degradation^9,12^. Daxx was shown to repress adenoviral replication by binding to α thalassemia/mental retardation syndrome X-linked (ATRX), and replication was restored via mutations that abolished the interaction of Daxx with ATRX^10^. The interaction between Daxx and heat shock protein 27 (HSP27) was confirmed by two-hybrid screening and glutathione S-transferase (GST) pull-down assays^13,14^. According to Charette et al.^13^, the phosphorylated dimeric form of HSP27, but not the unphosphorylated multimeric form of HSP27, interacts with Daxx both in vitro and in vivo, thereby preventing (a) the association of Daxx with FS-7-*a*ssociated *s*urface antigen (FAS) and ASK1 and (b) the induction of apoptosis by Daxx coexpressed with ASK1. In addition, Daxx interacts with the promyelocytic leukemia (PML) protein^15^ and the transcription factors p53, ETS1, PAX3, NF-κB, and E2F1^16^; however, the precise function of Daxx in cell death is controversial because conflicting results have been obtained from transient overexpression and gene knockout studies^17^.

The molecular chaperone HSP27 is a key regulator of protein folding and degradation in human cells^18^. Typically, HSP27 expression is very low; however, various stressful conditions such as heat shock, oxidative stress, inflammation, and exposure to cytotoxic agents or radiotherapy induce the massive accumulation of HSP27^19,20^. The stable overexpression of HSP27 has been observed in several human cancers, and high levels of HSP27 are associated with aggressive tumor phenotypes and poor prognosis^19^. Murine HSP25, which has biological functions similar to those of its human homolog, HSP27^21^, mediates the interaction between sirtuin 1 (SIRT1) and p53 and increases the deacetylation of p53, consequently decreasing apoptosis^22^.

In this study, we demonstrate that adenoviral replication in mouse cancer cells increases after Daxx downregulation or HSP25 induction by mechanisms different from those in human cancer cells.

## Materials and methods

### Cell lines and cell culture

Various human cancer cell lines (DU145, A375, MiaPaCa-2, HPAC, Huh7, Hep3B, MDAMB-231, and U251N cells) in addition to 293 A cells were cultured in Dulbecco’s modified Eagle’s medium (DMEM) with 10% fetal bovine serum (HyClone, Logan, UT, USA). Various mouse cancer cell lines (4T1, Panc02 and BNL 1ME A.7 R.1 cells) in addition to NIH3T3 cells were also cultured in DMEM with 10% fetal bovine serum, with the exception of B16BL6 cells, which were cultured in minimum essential medium (MEM). BNL 1ME A.7 R.1 (BNL) cells derived from BALB/c mice were kindly provided by Dr. M.L. Chen (National Taiwan University, Taiwan). Panc02 cells were kindly provided by Dr. N. Mayorek (The Hebrew University, Israel). All cells were maintained at 37 °C in a humidified atmosphere containing 5% CO_2_. To develop a stable BNL-CAR cell line, BNL cells were infected with retroviral LNCX-CAR. The infected cells were selected by using G418 (2 mg/ml). To generate a recombinant retrovirus based on LNCX, the Plat-A retrovirus packaging cell line (Cell Biolabs, Inc., San Diego, CA, USA), an amphotropic packaging cell line, was transfected with LNCX-CAR. LNCX-CAR was constructed by incorporating *Hin*dIII/*Cla*I-digested CAR after its production by PCR using pcDNA3.1hygro-CAR as the template. The construction of pcDNA3.1hygro-CAR was described in detail by Kang et al.^23^. LNCX-E1B55K was constructed by incorporating *Hpa*I/*Cla*I-digested E1B55K after its production by PCR using pcDNA3.1hygro-E1B55K, which was also described in detail by Kang et al.^23^. A stable B16BL6 cell line expressing CAR was selected by using puromycin (1 μg/ml), and CAR expression was confirmed after infection with retrovirus produced by transfecting pMXs-puro-CAR into the Plat-A packaging cell line. pMXs-puro-CAR was constructed by incorporating the CAR gene into BamHI-blunt-XhoI-digested pMXs-puro after the digestion of pcDNA3.1hygro-CAR with HindIII-blunt-XhoI. To establish B16BL6-CAR-E1B55K cells, B16BL6-CAR cells were infected with retroviral LNCX-E1B55K and selected by using G418 (2 mg/ml). A stable Panc02 cell line expressing myrAkt was selected by using puromycin (1.5 μg/ml), and myrAkt expression was confirmed after infection with retrovirus produced by transfecting pBabe-puro-myrAkt into the Plat-A packaging cell line. The BNL-HSP25, B16BL6-HSP25 and Panc02-HSP25 cell lines were obtained by transfection of the BNL, B16BL6, and Panc02 cell lines, respectively, with pcDNA3.1hygro-HSP25 and selected with hygromycin (250 μg/ml).

### Construction of Daxx-specific shRNAs

To construct human Daxx-specific shRNA, we screened five candidate sequences. Target selection was performed using an algorithm developed by Genolution Pharmaceuticals, Inc. (Seoul, South Korea). The selected target sequence was 5′-GCTACAAGCTGGAGAATGAGAAGCT-3′, and the loop sequence was 5′-TCTC-3′. To express human Daxx-specific shRNA in adenovirus, the top strand (5′-GATCCGCTACAAGCTGGAGAATGAGAAGCTTCTCAGCTTCTCATTCTCCAGCTTGTAGCTTTTTTA-3′) and the bottom strand (5′-AGCTTAAAAAA GCTACAAGCTGGAGAATGAGAAGCTGAGAAGCTTCTCATTCTCCAGCTTGTAGC G-3′) were annealed and subcloned into BamHI/HindIII-digested pSP72ΔE3-H1- shhTGFβ2. Mouse Daxx-specific shRNA was constructed in a similar manner; the target sequence was 5′-GTTAGGAAACAGCTATATAAAAGAA-3′, and the loop sequence was 5′-TCTC-3′. To express mouse Daxx-specific shRNA in adenovirus, the top strand (5′-GATCCGTTAGGAAACAGCTATATAAAAGAATCTCTTCTTTTATATAGCTGTTTCCTAACTTTTTTA-3′) and the bottom strand (5′-AGCTTAAAAAAGTTAGGAAACAGCTATATAAAAGAAGAGATTCTTTTATATAGCTGTTTCCTAACG-3′) were annealed and subcloned into BamHI/HindIII-digested pSP72ΔE3-U6-shNC (negative control) to yield pSP72ΔE3/U6-shmDaxx.

### Cloning of mouse HSP25

HSP25-expressing pcDNA3.1hygro was constructed for stable transfection into mouse liver cells or mouse melanoma cells. To clone mouse HSP25, RNA was extracted from BNL cells after heat shock at 43 °C for 4 h, followed by incubation at 37 °C for 24 h. HSP25 cDNA was generated via reverse transcription using reverse transcriptase and subsequent PCR using the following primers: sense primer, 5′-CGCGGATCCATGACCGAGCGCCGCGTGCC-3′, and antisense primer, 5′-CCGCTCGAGCTACTTGGCTCCAGACTGTT-3′. The BamHI/XhoI-digested PCR product was inserted into BamHI/XhoI-digested pcDNA3.1hygro to yield pcDNA3.1hygro-HSP25.

### Recombinant oncolytic adenovirus expressing human or mouse Daxx-specific shRNAs

To express human Daxx-specific shRNA by oncolytic adenovirus, the adenoviral shuttle vector pSP72ΔE3-H1-shhDaxx was linearized by XmnI. The adenoviral backbone vector dl324-BstBI was linearized by SpeI. The two linearized vectors were cotransformed into *E. coli* BJ5183 cells for the first homologous recombination. The resultant dl324-BstBI-H1-shhDaxx vector was linearized by Bsp1191, and pVAX1-3484-CMV-ΔE1B, a shuttle vector with replication competence, was linearized by PmeI. The construction of pVAX1-3484-CMV-ΔE1B was described in detail by Kim et al.^24^. The two linearized vectors were cotransformed into *E. coli* BJ5183 cells for the second homologous recombination to yield dl324-3484-ΔE3-H1-shhDaxx (Ad-3484-shhDaxx). To express mouse Daxx-specific shRNA from oncolytic adenovirus, pSP72ΔE3-U6-shmDaxx was used as a shuttle vector, and the process was repeated in the same manner for human Daxx.

### Construction of the 5′-flanking region of the mouse Daxx gene

We searched the mouse Daxx promoter region from mouse genomic DNA originating from EBI Database accession No. AF110520.1. First, the promoter region of Daxx was sequenced using a primer (5′- GTCTCCGTCTTACACAGTTC-3′) that binds near the N-terminal Daxx coding sequence from BNL (or B16BL6) genomic DNA and aligned with the human Daxx promoter sequence provided by Li et al.^25^. As a result, a 659 bp fragment in this region similar to the human Daxx promoter region spanning from −659 to −1 was generated by PCR using the following primers: forward, 5′- TGCTGTGCTCATTTGTATGCG-3′, and reverse, 5′-CATAGTTCCCTCCGCCTTCC-3′. For PCR, BNL genomic DNA was used as a template. To confirm the mouse Daxx promoter sequence, the PCR product was subcloned into T-vector pMD20 (TaKaRa, Japan), which has a dT overhang at the 3′ end, and sequenced (Fig. 5a).

### Construction of Daxx promoter-luciferase reporter plasmids

To construct mouse Daxx promoter-luciferase reporter plasmids, a 659 bp fragment spanning from −659 to −1 was generated by PCR using the following primers: 5′-CGGTGGTACCTGCTGTGCTCATTTGTATGC-3′ and 5′-ATCTAAGCTTTTCCTCTCCCCAACCCCCAC-3′, which contain the KpnI and HindIII restriction enzyme sites, respectively. PCR constructs were then subcloned into the pGL3-basic luciferase vector (Promega, Madison, WI, USA) at the KpnI and HindIII sites to produce a full length Daxx-p 659 construct. Furthermore, to produce putative Daxx promoter-luciferase constructs, a series of 5′-3′ or 3′-5′ Daxx-deleted promoter constructs, Daxx-p 479, 299, 159, 500, and 590, were generated by PCR using their corresponding PCR primer pairs and then ligated with the pGL3-basic vector. The Daxx-p 69 deletion mutant construct was constructed after annealing the following two strands and ligating them with the pGL3-basic vector: 5′-CGTGCTCCAGGCGGAAGCGCTAAGGCTTCCGGTCTGTTGTGGGGTCTGCGGTGGGGGTTGGGGAGAGGAA-3′ and 5′- AGCTTTCCTCTCCCCAACCCCCACCGCAGACCCCACAACAGACCGGAAGCCTTAGCGCTTCCGCCTGGAGCACGGTAC-3′. The mutant Daxx-p159 construct, in which one base in the Sp1-binding site was mutated (GGGCGAG → GGGCGGG), in the pUC57 vector was provided by Bionics (Seoul, South Korea) and cloned into the pGL3-basic vector.

### Construction of stat3 promoter-luciferase reporter plasmids

The mouse stat3 promoter sequence was provided by Ichiba et al.^26^. To confirm the stat3 promoter sequence in the mouse BNL cell line, the stat3 promoter region was obtained from BNL cell genomic DNA by PCR using the following primers: 5′-TTAAGTGGGGTGACACCTGGG-3′ and 5′- CAGGTTCCCCCTCCCTGTCTA-3′. The resultant PCR product from −476 to 62 bp was subcloned into the pMD20-T vector and sequenced. The sequence of the PCR product is provided in Supplementary Fig. 2. For the luciferase assay, this PCR product in the pMD20-T vector was subcloned into the pGL3-basic luciferase vector at the *Kpn*I and *Hind*III sites to produce a mouse stat3 promoter construct.

### Chromatin immunoprecipitation (ChIP) assay

A CHIP assay was performed to analyze the ability of the stat3 protein to bind the Daxx promoter using a Pierce Magnetic ChIP kit (Thermo Scientific, Rockford, IL, USA) according to the manufacturer’s instructions. The following primer pairs were used to amplify the Daxx core promoter (−161 to −1):

Human Daxx ChIp assay primer

- Forward: TGAAATCCCCACCACTTCCTCCCTC
- Reverse: GAGAGGCAGTGTTTTCAGCATTTGT

Mouse Daxx ChIp assay primer

- Forward: CAGGTGCACACACACACGAACACAC
- Reverse: CATAGTTCCCTCCGCCTTCCTCTCC

### Construction of various HSP25 proteins substituted with the HSP27 domain

The 2 most variable amino acid regions of HSP25 compared to those of HSP27 were substituted with the corresponding HSP27 amino acids. The 1st region was amino acids 47–51 of HSP25 (FSAAG), which was substituted with amino acids 46–50 of HSP27 (LGGSS). To change the amino acids in box I, the DNA sequence was changed from ttcagcgccgctggg (FSAAG) to ttaggcggcagcagc (LGGSS). To substitute box I of HSP25 with the corresponding sequence of HSP27, the 5′-HindIII to 3′-SacI HSP25 fragment containing HSP27 ttaggcggcagcagc (LGGSS), which originated from pUC57-chimeric HSP25, was inserted into *Hind*III/*Sac*I-digested pcDNA3.1hygro-HSP25. To produce various substituted HSP25 proteins, the *Sac*I site in the CMV promoter of pcDNA3.1 was deleted by site-directed mutagenesis with sense primer (5′-TATATAAGCAGAGCTGTCTGGCTAACTAGAG-3′) and antisense primer (5′-CTCTAGTTAGCCAGACAGCTCTGCTTATATA-3′). To produce pcDNA3.1hygro-HSP25 without *Hind*III at the multicloning site, *Bam*HI-blunt/XhoI-digested HSP25 was inserted into NheI-blunt/XhoI-digested pcDNA3.1hygro. Box II, which consisted of amino acids 61–72 of HSP25 (AATAEGPAAVTL), was substituted with amino acids 60-68 of HSP27 (PAAIESPAV). To substitute box II of HSP25 with the corresponding HSP27 sequence, a 5′-HindIII to 3′-SacI HSP25 fragment containing HSP27 cccgccgccatcgagagccccgcagtg (PAAIESPAV), which originated from pUC57-chimeric HSP25, was inserted into HindIII/SacI-digested pcDNA3.1hygro-HSP25. To substitute both box I and box II of HSP25 with the corresponding HSP27 sequences, a 5′-HindIII to 3′-SacI HSP25 fragment containing box I of HSP27, ttaggcggcagcagc (LGGSS), and box II of HSP27, cccgccgccatcgagagccccgcagtg (PAAIESPAV), which originated from pUC57-chimeric HSP25, was inserted into HindIII/SacI-digested pcDNA3.1hygro-HSP25.

### Animal studies

The animal protocol (2016-0014) used in this study was reviewed and approved by the Institutional Animal Care and Use Committee of the Yonsei University Health System. To generate a xenograft tumor model, 6 × 10^6^ MiaPaCa-2 cells or 1 × 10^7^ Panc02-myrAkt cells were injected with Corning Matrigel Matrix (Corning, Corning, NY, USA) into the subcutaneous abdominal regions of male BALB/c nude mice or C57BL/6 mice, respectively. The Panc02-myrAkt cells injected in the mice were a pool of selected clones (#2, #5, #7, #9, #10) (Supplementary Fig. 8). When the tumors derived from MiaPaCa-2 cell or Panc02-myrAkt cell reached an average size of 50–60 mm^3^ or 80–100 mm^3^, respectively, the mice were randomized into three groups (PBS, Ad-3484-shNC, Ad-3484-shDaxx) of 5 mice each. The oncolytic adenoviruses used were oncolytic negative control (NC) adenovirus (Ad-3484-shNC) and human or mouse Daxx-specific shRNA-expressing oncolytic adenovirus (Ad-3484-shDaxx). The mice in each group received intratumoral injections of 1 × 10^9^ plaque forming units (pfu) of one of the two oncolytic adenoviruses diluted in 50 μl of PBS or PBS alone. Intratumoral injections were repeated every other day for a total of three injections. Tumor growth was measured using a caliper every 2 days for 15 days until necrotic cell death was observed. Tumor volume (V) was calculated using the following formula: V (mm^3^) = 0.52 × length (mm) × width (mm)^2^.

### Immunohistochemistry

Seven days after the subcutaneous injection of MiaPaCa-2 or Panc02-myrAkt cells into the abdominal regions of male BALB/c nude mice or C57BL/6 mice, respectively, one of two oncolytic adenoviruses (Ad-3484-shNC or Ad-3484-shDaxx, 1 × 10^9^ pfu/50 μl) was intratumorally injected every other day for a total of three injections. Seven days after the last viral injection, tumor tissues were extracted, fixed for 24 h in 10% formaldehyde, and paraffin embedded for immunohistochemical (IHC) staining.

### Statistical analyses

Data are presented as the mean ± standard error of the mean (S.E.M.). Differences between groups were examined using unpaired two-tailed t-tests. *P* values were calculated using GraphPad Prism version 6.0. *P* < 0.01 or *P* < 0.05 indicated statistical significance. All experiments were performed three times independently.

## Results

### Silencing of Daxx in murine and p53-mutant human cancer cells increases adenoviral replication

**Fig. 1:**
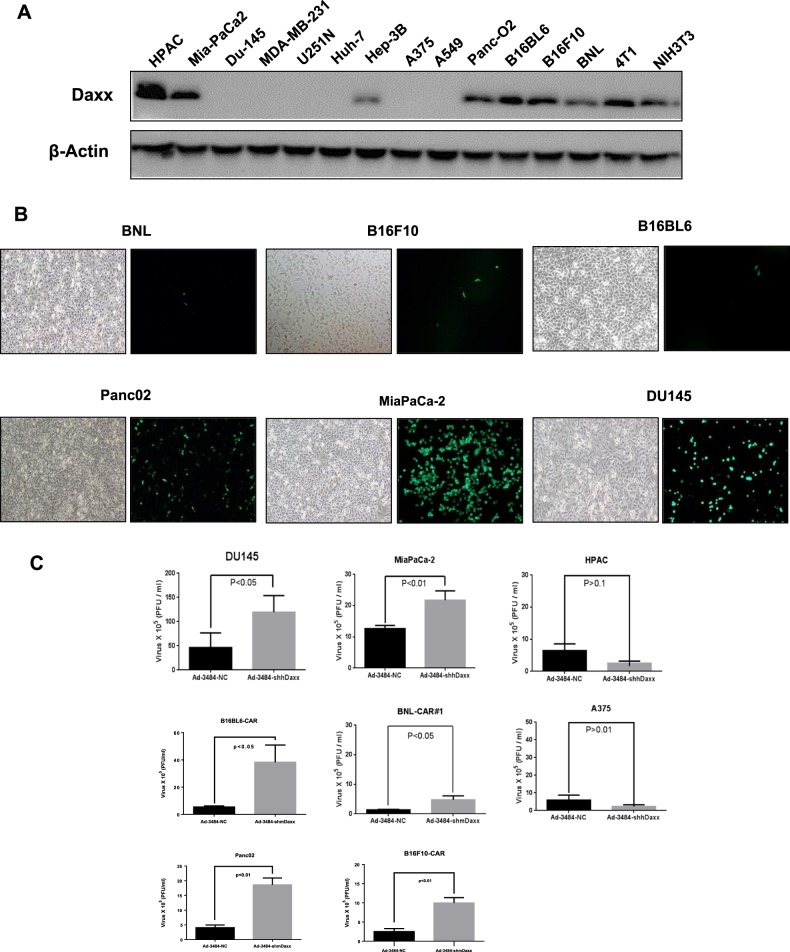

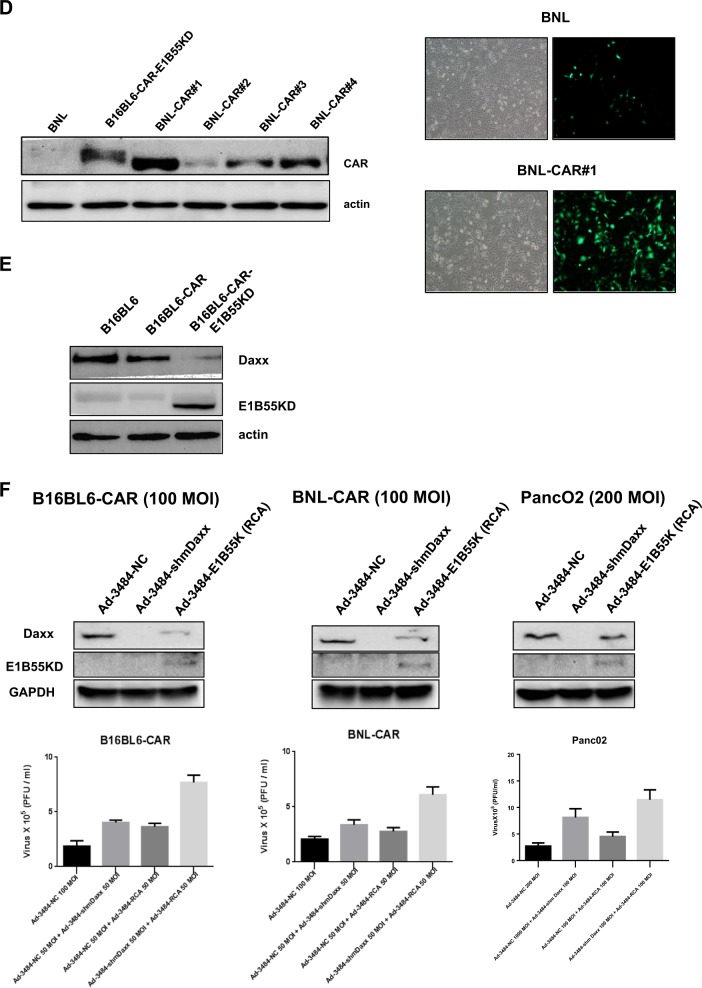

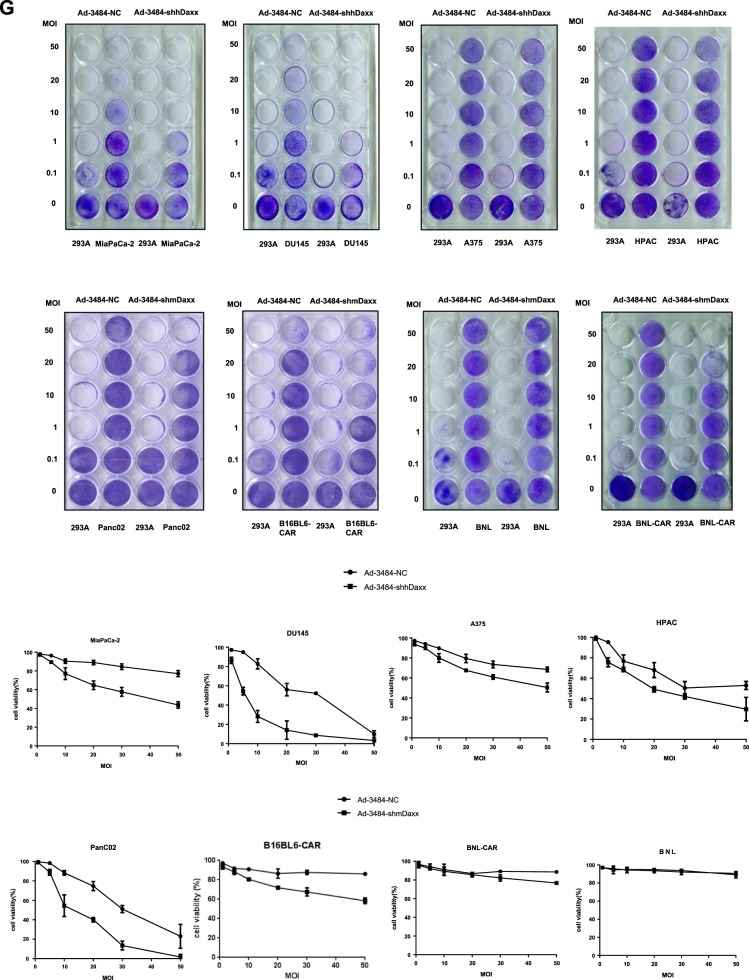
Enhanced adenoviral replication by silencing Daxx in mice. **a** Cellular Daxx levels in human cancer cell lines (left), mouse cancer cell lines and two human cancer cell lines used as controls (right) were examined by immunoblotting. **b** Adenoviral infectivity of various mouse and human cancer cell lines was examined using replication-incompetent GFP-expressing adenovirus after 48 h of infection (multiplicity of infection [MOI] of 20; ×100 magnification). **c** Adenoviral replication was enhanced by Daxx downregulation in mouse or p53-mutant human cancer cell lines using Daxx-specific shRNA-expressing oncolytic adenovirus at an MOI of 100 for 48 h. Panc02 and B16F10-CAR cells were infected at an MOI of 200 for maximal infectivity. The same number of all cancer cell lines (1 × 10^5^ cells) were plated onto 6-well plates and infected with Ad-3484-NC or Ad-3484-shDaxx at an MOI of 100 or 200 for maximal infectivity. After 48 h of infection, each viral soup (3 ml) was titrated after repetitive freeze-thaw cycles. Ad-3484-shhDaxx (shhDaxx, shRNA targeting human Daxx), Ad-3484-shmDaxx (shmDaxx, shRNA targeting mouse Daxx), and Ad-3484-NC (NC, negative control, the scrambled DNA sequence of the shRNA). Ad-3484-NC adenovirus is a replication-competent adenovirus used as a negative control, and 3484 indicates the presence of E1A in the viral genome controlled by the cytomegalovirus (CMV) promoter lacking the E1B region. Error bars represent standard errors from three independent experiments. **d** BNL mouse cell lines were infected with LNCX-CAR, and a clone expressing CAR was selected for efficient adenoviral replication in mouse cell lines (left). Enhanced adenoviral infection was observed in clone #1 of BNL-CAR after infection with replication-incompetent GFP-expressing adenovirus at an MOI of 20 for 48 h (right, 100 × magnification). **e** Cellular Daxx levels were examined in B16BL6 cells, CAR-expressing B16BL6 cells (B16BL6-CAR) and B16BL6 cells expressing both CAR and E1B55K (B16BL6-CAR-E1B55K) by immunoblotting. **f** Cellular Daxx levels and viral production were examined after infection with oncolytic adenovirus expressing shRNA targeting mouse Daxx without E1B55K and/or RCA expressing E1B55K at an MOI of 50 or 100 for 48 h. **g** To compare the oncolytic activity induced by Ad-3484-NC with that induced by Ad-3484-shhDaxx or Ad-3484-shmDaxx, human and mouse cancer cell lines were infected with each virus at an MOI between 0.1 and 50. Immediately after 293 A cells infected with one of the viruses at an MOI of 0.1 exhibited complete cell lysis, all remaining cells on the plate were fixed with 4% paraformaldehyde and stained with 0.5% crystal violet (upper). Cell viability after infection with each virus was compared by cell death curves in a quantitative graph (lower). Error bars in **a**, **c**, **e**, and **g** represent standard errors from three independent experiments

Adenoviral replication is effectively maintained only when the adenoviral E1B55K protein binds to Daxx and induces its proteasome-dependent degradation^9^. Previously, we showed that Daxx downregulation enhanced adenoviral replication^11^. In this study, we examined cellular Daxx levels in various human and mouse cancer cell lines. Interestingly, cellular Daxx levels were higher in all examined mouse cancer cell lines than in all examined human cancer cell lines (Fig. 1a). Therefore, we speculated that low adenoviral replication in mouse cells is caused by increased cellular levels of Daxx, which represses adenoviral replication^11,12^. We then examined adenoviral infectivity in mouse and human cancer cells. The mouse cancer cell lines examined, except Panc02 cells, showed very low infectivity (Fig. 1b, Supplementary Fig. 1). However, although infectivity in B16BL6, B16F10 or BNL mouse cancer cells increased to a level similar to that in human cancer cells with the introduction of coxsackievirus and adenovirus receptor (CAR) (Supplementary Fig. 2), viral production in B16BL6-CAR, B16F10-CAR, BNL-CAR, and Panc02 cells was at least 10-fold lower than that in DU-145 and MiaPaCa-2 cells (Fig. 1c). Downregulation of cellular Daxx in mouse cancer cell lines (B16BL6-CAR, B16F10-CAR, BNL-CAR, and Panc02 cells) and p53-mutant human cancer cell lines (MiaPaCa-2 and DU145 cells) by infection with Daxx-specific shRNA-expressing oncolytic adenovirus increased adenoviral replication (Fig. 1c). In contrast, the replication of Daxx-specific shRNA-expressing oncolytic adenovirus was not increased in human cancer cell lines expressing wild-type p53 (HPAC and A375 cells; Fig. 1c). In BNL cells with very low adenoviral infectivity, the introduction of CAR-expressing retrovirus increased infectivity by more than 5-fold (Fig. 1d). E1B55K expression in the form of replication-competent adenovirus (RCA) did not significantly increase viral production compared to that observed with Daxx-shRNA expression in the oncolytic adenovirus without E1B55K (Fig. 1e). In fact, E1B55K in the RCA decreased Daxx levels to a lesser extent than the expression of Daxx-shRNA in the adenovirus (Fig. 1fa), and additional Daxx downregulation with RCA significantly increased viral production (Fig. 1fb), which suggests that Daxx downregulation plays a critical role in enhancing viral production in even E1B55K-containing oncolytic adenovirus. This finding provides a strong impetus to utilize oncolytic adenoviruses with reduced Daxx expression regardless of E1B55K expression. Furthermore, decreased cellular Daxx expression increased oncolytic activity in both mouse and mutant p53-harboring human cancer cell lines (Fig. 1g), which was similar to the pattern of virus production observed in murine cancer cell lines (Fig. 1c).

### Mechanisms by which the proteasomal degradation of Daxx is regulated differ between human and murine cancer cells

**Fig. 2:**
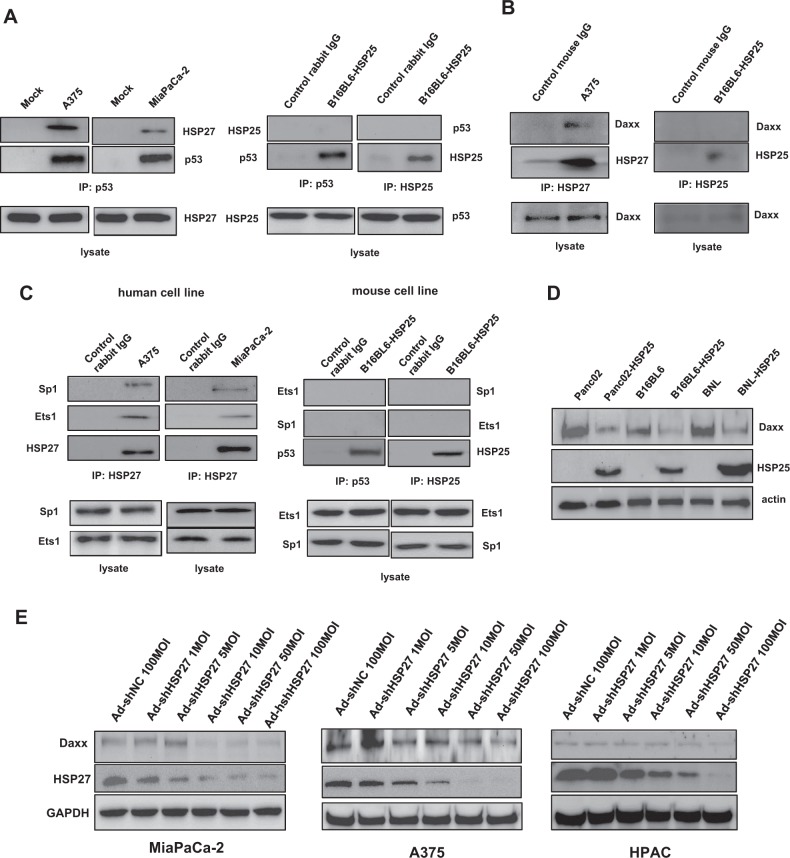

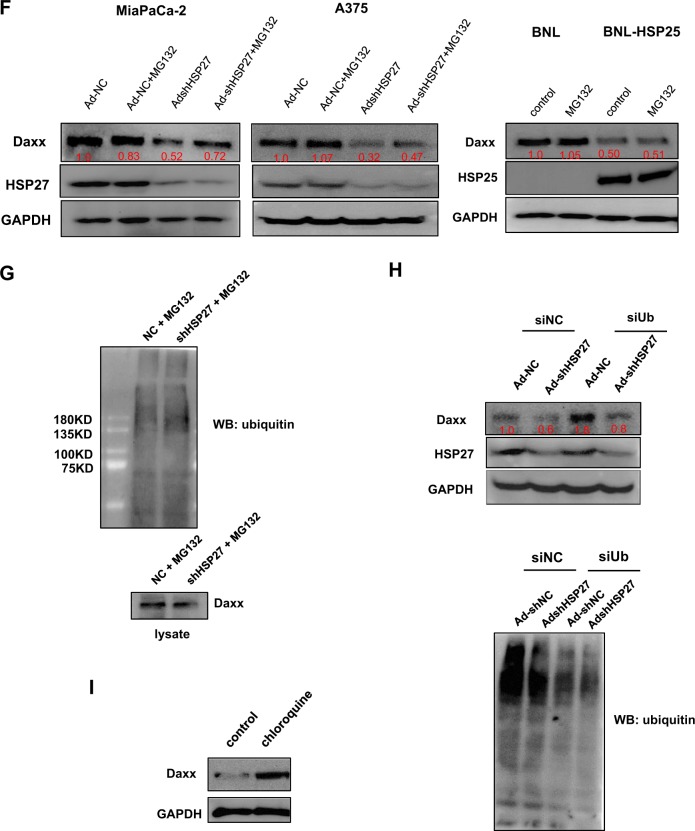
Comparison of proteasomal Daxx degradation in human and mouse cells. **a** Human A375 and MiaPaCa-2 cells and mouse B16BL6-HSP25 cells were lysed and subjected to immunoprecipitation with an anti‐p53 (left, right) or anti-HSP25 (right) antibody to detect the interaction between p53 and HSP27 (or HSP25). **b** Human A375 and MiaPaCa-2 cells and mouse BNL-HSP25 cells were lysed and subjected to immunoprecipitation with an anti‐HSP27 (or anti-HSP25) antibody to detect the interaction between HSP27 (or HSP25) and Daxx. **c** Human A375 and MiaPaCa-2 cells and mouse BNL-HSP25 cells were lysed and subjected to immunoprecipitation with an anti‐p53 or anti-HSP27 (or anti-HSP25) antibody to detect the interaction between p53 and Ets1/Sp1 or HSP27 (or HSP25) and Ets1/Sp1. **d** Cellular Daxx levels in various mouse cancer cell lines were examined after the stable transfection of PancO2, B16BL6 and BNL cells with pcDNA3.1hygro-HSP25. **e** Validation of HSP27 downregulation-dependent Daxx downregulation in human cancer cells after infection with defective adenovirus expressing shRNA against HSP27 at MOIs between 1 and 100. Then, cellular Daxx and HSP27 levels in human cancer cells were detected by western blotting. **f** Cellular Daxx levels in human (MiaPcCa-2, A375) and mouse (BNL) cancer cell lines treated with MG132 (10 µM, 4 h) after infection with defective adenovirus expressing shRNA against HSP27 (48 h) or stably expressing HSP25 were examined. The numbers indicate the band intensity relative to that of Ad-NC (MiaPaCa-2, A375 cells) or control (BNL cells) after band intensities were measured with a densitometer. **g** Human MiaPaCa-2 cells after infection with defective adenovirus expressing shRNA targeting HSP27 at an MOI 100 treated with MG132 (10 µM, 4 h) were lysed and subjected to immunoprecipitation with an anti-Daxx antibody to detect ubiquitin. **h** Human MiaPaCa-2 cells were transfected with NC siRNA (negative control, scrambled siRNA) or siRNA against ubiquitin, followed by infection with defective adenovirus expressing NC shRNA NC or shRNA against HSP27 (MOI of 100, 48 h). Cellular levels of Daxx were examined to determine whether HSP27 downregulation-mediated Daxx degradation is ubiquitin-dependent (upper), and cellular levels of ubiquitinated proteins were detected (lower). The numbers indicate the band intensity relative to that of siNC/Ad-NC after band intensities were measured with a densitometer. **i** MiaPaCa-2 cells were treated with chloroquine (30 µM, 6 h), and cellular levels of Daxx were examined

The importance of p53 status for viral replication in human and mouse cells was further investigated by introducing mutant p53 into human and mouse cancer cells that harbor endogenous wild-type p53. Unlike the increase in viral production observed after the introduction of mutant p53 in human cells with wild-type p53, the introduction of mutant p53 into mouse cancer cell lines with wild-type p53 did not increase viral replication. In contrast, Daxx downregulation increased viral production in mice irrespective of p53 status or cell type (Supplementary Fig. 3). To better understand why the murine cancer cell lines expressed higher levels of Daxx at resting state than the human cancer cell lines, we examined the expression levels of HSP27 and its murine homolog, HSP25, in the human and murine cancer cell lines, respectively, because HSP27 has been previously shown to be a common binding partner of both p53 and Daxx in human cells^27^. First, interactions between these HSPs and p53/Daxx in our cell lines were verified using coimmunoprecipitation assays (Fig. 2a, left). The interaction between p53 and HSP27 was observed in human cancer cells; however, unexpectedly, HSP25 was not detected in any of the examined mouse cancer cell lines (data not shown), and no interaction between p53 and HSP25 was found (Fig. 2a, right). Similar results were also observed for the interactions between Daxx and HSP27 or HSP25 (Fig. 2b, left, right). In addition, HSP27 interacted with the transcription factors ETS1 and Sp1, but HSP25 did not interact with ETS1 and Sp1 (Fig. 2c). Interestingly, cellular Daxx levels were markedly decreased upon the introduction of HSP25 into mouse cancer cells (Fig. 2d). In contrast, HSP27 downregulation in various human cancer cell lines decreased Daxx expression in a dose-dependent manner (Fig. 2e). The decrease in Daxx levels after HSP27 downregulation in human cancer cells was at least in part due to the proteasomal degradation of Daxx (Fig. 2f, left); in contrast, the decrease in Daxx levels in mouse BNL cells overexpressing HSP25 was not related to proteasomal degradation (Fig. 2f, right). The mechanisms by which Daxx undergoes proteasomal degradation after HSP27 downregulation were further evaluated by analyzing the ubiquitination of Daxx in human MiaPaCa-2 cells. Unexpectedly, however, an increase in ubiquitinated Daxx was not observed upon HSP27 downregulation (Fig. 2g). Accordingly, the downregulation of ubiquitin by siRNA did not lead to a further reduction in Daxx protein levels with HSP27 shRNA treatment (Fig. 2h). Thus, the proteasomal degradation of Daxx induced by HSP27 downregulation is ubiquitin-independent. Meanwhile, treatment with siRNA targeting ubiquitin enhanced the basal level of Daxx in control Adeno-shNC-infected cells (Fig. 2h), and this increase in the basal level of Daxx was also observed when lysosomal degradation was inhibited by chloroquine treatment (Fig. 2i). These findings indicate that Daxx degradation at the stationary phase occurs through a ubiquitin- and lysosome-dependent mechanism. Taken together, these data demonstrate that HSP27 functions as a molecular chaperone to protect cellular Daxx from proteasomal degradation in human cancer cells, but HSP25 does not play a similar role in murine cancer cells, at least with respect to mouse Daxx. Another possibility is that HSP25 functions as an inhibitor of Daxx transcription.

### HSP25 localizes predominantly to the cytoplasm of murine cells, where it impairs the nuclear localization of positive regulator(s) of Daxx transcription

**Fig. 3:**
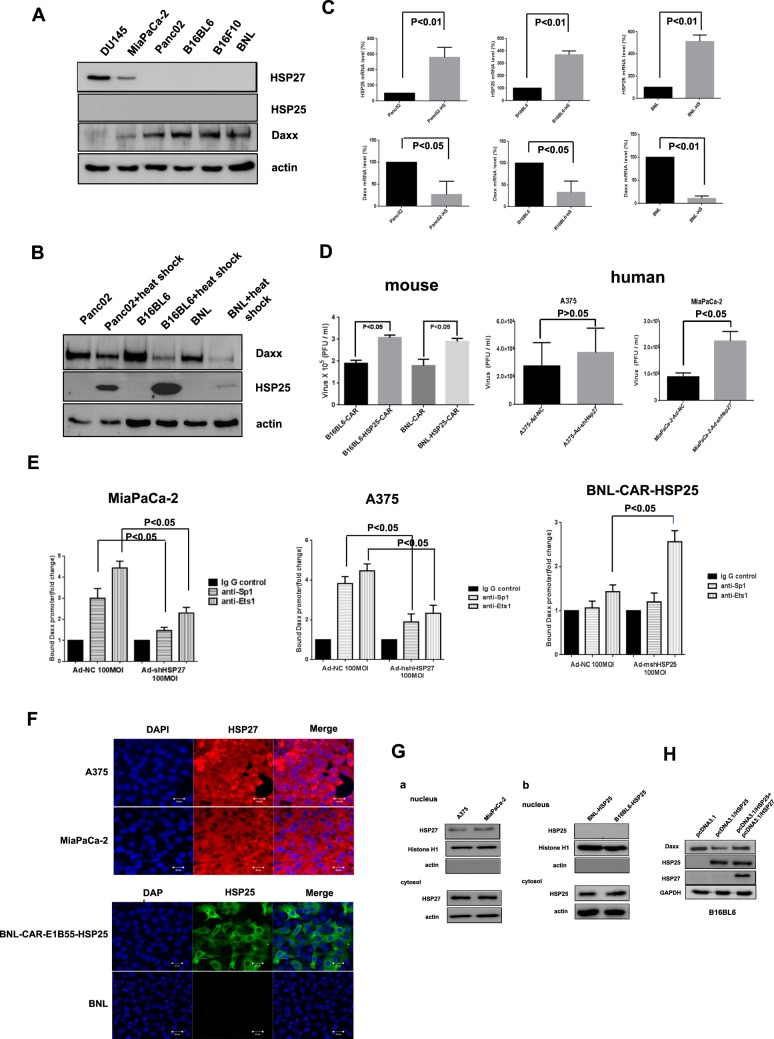
HSP25 is a cellular counterpart of adenoviral E1B55K in mice. **a** To investigate the correlation between Daxx and HSP27 (or HSP25), cellular HSP27, HSP25, Daxx, and actin levels were examined by western blot analysis. **b** Cellular Daxx levels in various mouse cancer cell lines that underwent heat shock induction of HSP25 were examined at 43 °C for 4 h followed by incubation at 37 °C for 24 h. **c** Cellular Daxx and HSP25 mRNA levels were examined in various mouse cancer cells by real-time RT-PCR after heat shock at 43 °C for 4 h followed by incubation at 37 °C for 24 h. HS: heat shock **d** Enhanced adenoviral replication was confirmed in mouse cancer cells stably expressing HSP25 or human cancer cells in which HSP27 was downregulated. After infection of B16BL6-CAR or BNL-CAR cells stably expressing HSP25 with oncolytic adenovirus at an MOI of 100 (Ad-3484-NC) for 48 h, viral production was estimated (left). After infection of human cancer cells with oncolytic adenovirus at an MOI of 100 (Ad-3484-NC or Ad-3484-shHSP27) for 48 h, viral production was estimated (right). **e** Human MiaPaCa-2 and A375 cells and mouse BNL-CAR-HSP25 HPAC cells were infected with defective adenovirus expressing shRNA against HSP27 (or HSP25) at an MOI of 100. After 48 h, Daxx promoter activity was analyzed by Chip assays using antibodies against SP1 or Ets1. **f** The distribution of HSP27 or HSP25 was examined using confocal immunofluorescence. Cellular HSP27 or HSP25 was detected with species-specific primary anti-HSP27 or anti-HSP25 antibodies conjugated to Flamma 552 or Flamma 488, respectively. **g** The cytoplasmic/nuclear localization of HSP27 or HSP25 was examined in the cytoplasm and nuclei of human **a** or mouse cancer cells **b** after their fractionation using cytoplasmic (actin) and nuclear (histone H1) marker proteins, respectively. **h** Recovery of Daxx expression with the introduction of HSP27 into mouse B16BL6 cells. Cellular Daxx levels were examined after B16BL6 cells were transfected with pcDNA3.1/HSP25 or pcDNA3.1/HSP25 + pcDNA3.1/HSP27. Error bars in **c**, **d**, and **e** represent standard errors from three independent experiments

Although neither HSP25 nor HSP27 were detected in mouse cancer cells, the cellular levels of Daxx were much higher in mouse cells than in human cancer cells (Fig. 3a), supporting the notion that HSP25 is not required as a molecular chaperone to protect Daxx from degradation in mouse cancer cells. Furthermore, cellular Daxx levels decreased markedly after the heat shock induction of HSP25 expression in these mouse cancer cells (Fig. 3b), and transcriptional repression of Daxx was triggered by the transcriptional induction of HSP25 in these cells, suggesting that Daxx expression is inversely proportional to HSP25 gene expression (Fig. 3c). Finally, adenoviral replication in both B16BL6-CAR and BNL-CAR cells increased markedly following HSP25 expression (Fig. 3d, left). In contrast, adenoviral production increased upon the downregulation of HSP27, especially in human cancer cells with mutant p53 (Fig. 3d, right). Next, we demonstrated that the decrease in Daxx levels following HSP27 downregulation in human cancer cells resulted from decreased binding of ETS1 and SP1 to the Daxx promoter (Fig. 3e, left, middle), whereas HSP25 downregulation in murine cells increased ETS1 binding to the Daxx promoter (Fig. 3e, right). Taken together, these data led us to hypothesize that most of the induced HSP25 was localized in the cytoplasm and that HSP25 strongly inhibited the nuclear translocation of positive regulator(s) of Daxx gene expression. Unlike the arginine-rich nuclear localization signal (NLS) of *Drosophila* HSP27^28^, neither human HSP27 nor mouse HSP25 has an arginine-rich NLS. Therefore, we sought out potential NLSs and used the PSORT program to predict subcellular protein localization sites. HSP27 was predicted to localize to the nuclei (39.1%), cytoplasm (47.8%) and mitochondria (13.0) of human cells; in contrast, HSP25 was predicted to localize mainly outside the nucleus (77.8%), with only 22.2% of HSP25 localizing within the nucleus. Thus, the proportion of nuclear HSP25 is half that of HSP27. In fact, HSP25 in mouse cells was primarily located in the cytoplasm, whereas HSP27 was ubiquitously expressed in both the nucleus and cytoplasm (Fig. 3f, g). Thus, these findings supported our hypothesis that HSP25 likely sequesters positive regulator(s) of Daxx expression. We then examined whether the introduction of HSP27 into mouse cells would provide a shuttle to move these putative Daxx positive regulator(s) into the nucleus, leading to the recovery of cellular Daxx levels. As shown in Fig. 3h, cellular Daxx levels were significantly upregulated by HSP27 overexpression, which presumably enhanced the nuclear localization of putative positive regulator(s) of Daxx transcription.

### Absence of the Sp1-binding site in the murine Daxx promoter and identification of the Daxx core promoter region

**Fig. 4:**
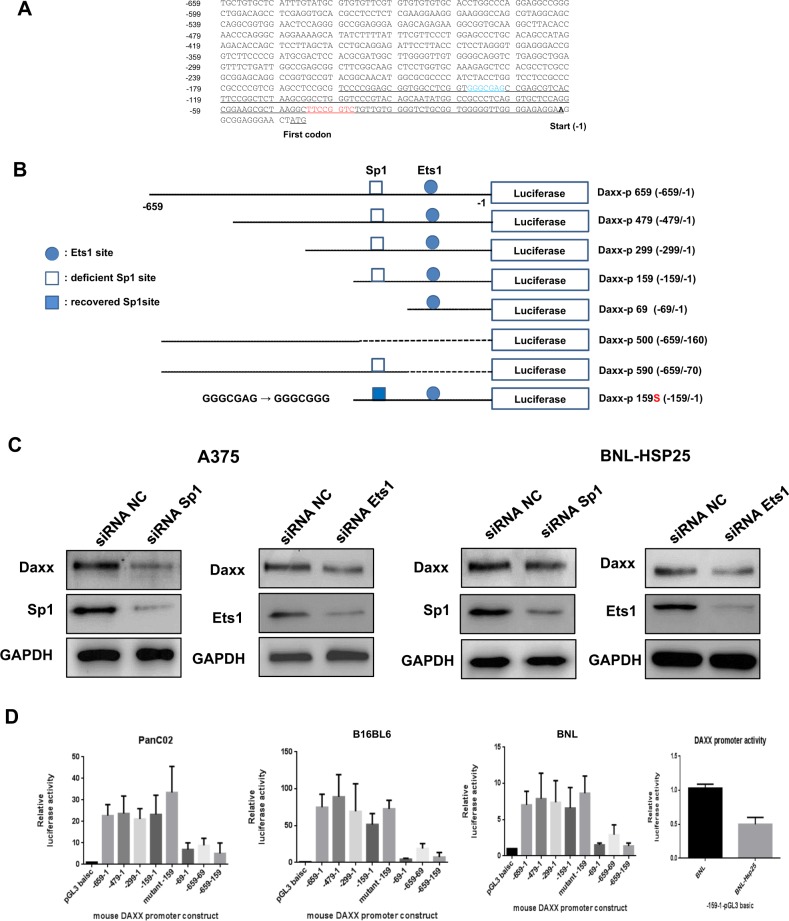
Characterization of the Daxx (death domain associated protein) promoter region in mouse cell lines. **a** The nucleotide sequence of the 5’-UTR of the Daxx gene from BNL cells contains Ets1 (red) and deficient Sp1 (blue) consensus sequences in the core promoter region (underlined). The first codon (bold, black) is indicated. **b** Schematic representation of Daxx promoter-luciferase reporter plasmids in mouse cells: Daxx-p659 containing the full-length promoter with a putative Ets1-binding site; Daxx-p479, Daxx-p299, Daxx-p159, Daxx-p69, Daxx-p500, and Daxx-p590 containing a series of 5’-3’ or 3’-5’ deleted promoters; Daxx-p159S, which is Sp1 site-recovered Daxx-p159 **c** Cellular levels of Daxx were examined after transfection of A375 or BNL-HSP25 cells with siRNA against Sp1 or Ets1. **d** Transient transfection of various mouse cell lines and luciferase assays to determine Daxx promoter activity. Cells were transfected with 1 μg of each Daxx promoter construct or the pGL3-basic vector, which served as a negative control. Data represent the mean ± standard error of three independent experiments normalized to the activity of Renilla luciferase with the CMV promoter (pRL) (cotransfection with pRL-CMV as an internal control). Error bars represent standard errors from three independent experiments

Unlike the positive correlation observed between the expression levels of Daxx and HSP27 in human cancer cells, Daxx levels were much higher in mouse cancer cells than in human cancer cells with concomitant undetectable levels of HSP25, as mentioned earlier (Figs. 1a, 2a). To determine whether there are any significant differences between the human and mouse Daxx promoters, the Daxx promoter region was analyzed. We found that the consensus Sp1 transcription factor-binding site was absent in the mouse Daxx promoter (Fig. 4a, b, Supplementary Fig. 7). Furthermore, cellular Daxx levels in mouse cells did not change following Sp1 downregulation (Fig. 4c), suggesting that Sp1 is not a critical driver of transcription from the Daxx promoter. Next, we examined the involvement of the Sp1-binding site in the human Daxx promoter in mouse cancer cells by changing the adenine in the murine Daxx promoter to guanine to mimic the human Sp1-binding sequence. The activity of the mutated Daxx promoter was enhanced compared with that of the wild-type core promoter in three different mouse cell lines (Fig. 4d). These experiments revealed that nucleotides −1 to −159 in the Daxx promoter region in mouse cancer cells can provide core promoter activity despite the lack of an Sp1-binding site.

### Signal transducer and activator of transcription 3 (stat3) acts as a positive regulator of Daxx expression

**Fig. 5:**
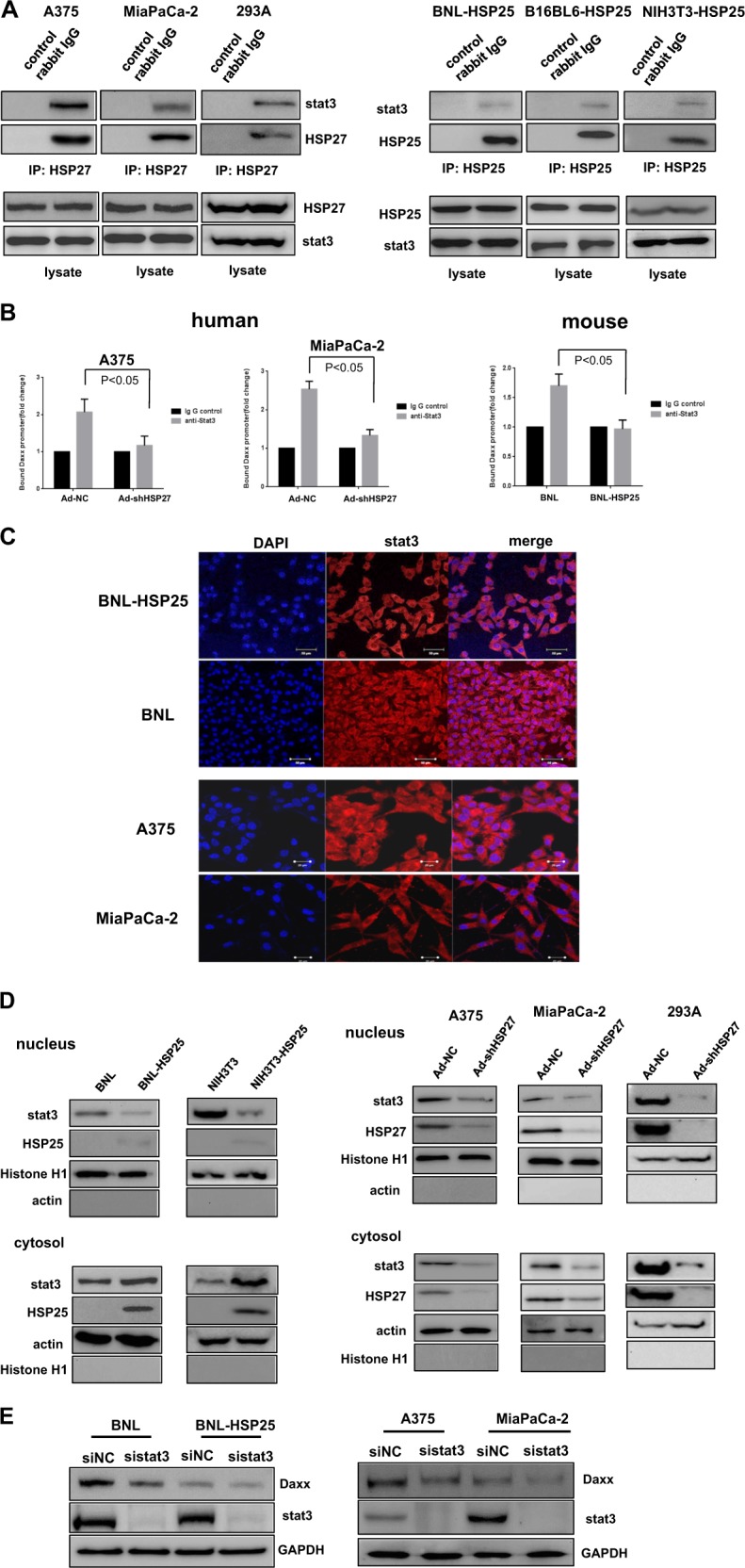
Stat3 binding to HSP27 or HSP25 positively regulates Daxx expression. **a** Human A375 and MIaPaCa-2 cells and mouse BNL-HSP25 and B16BL6-HSP25 cells were lysed and subjected to immunoprecipitation with anti-HSP27 (left) or anti-HSP25 antibodies (right) to detect the interaction between HSP27 (HSP25) and stat3. **b** Daxx promoter binding was analyzed by ChIp assays using antibody against stat3. BNL and BNL-HSP25 mouse cancer cells and A375 and MiaPaCa-2 human cancer cells infected with adenovirus (NC or shRNA against HSP27) at an MOI of 100 were used to immunoprecipitate stat3 to determine the effect of HSP25 or HSP27 on Daxx promoter binding. Error bars represent standard errors from three independent experiments. **c** The distribution of stat3 was examined using confocal immunofluorescence. Cellular stat3 was detected with species-specific primary anti-stat3 antibody conjugated to Flamma 552. **d** The cytoplasmic/nuclear localization of stat3 in a BNL mouse cell line with or without HSP25 was determined by cytoplasmic and nuclear fractionation followed by detection using cytoplasmic (actin) and nuclear (histone H1) marker proteins, respectively (left). Cytoplasmic/nuclear localization of stat3 in human cell lines after their infection with adenovirus (NC or shRNA against HSP27) at an MOI of 100 was determined by cytoplasmic and nuclear fractionation followed by detection using cytoplasmic (actin) and nuclear (histone H1) marker proteins, respectively (right)

Next, we sought to identify binding partners of HSP25 and HSP27 that might function as transcription factors to drive Daxx expression. According to Arrigo et al.^27^, the transcription factor stat3 is a potential HSP27-binding partner that also binds to the Daxx promoter^29^, as reported in the “GeneCards” database. Coimmunoprecipitation assays showed that stat3 interacts with HSP25 and HSP27 in mouse and human cells, respectively (Fig. 5a). Intriguingly, stat3 binding to the Daxx promoter region was significantly inhibited by HSP25 expression (Fig. 5b); this observation raised the possibility that HSP25 interferes with the ability of stat3 to bind to and activate transcription from the Daxx promoter, which stands in contrast to the role of HSP27 in promoting stat3-mediated Daxx transcription in human cells. This result was further confirmed by the observation of differential stat3 localization in the nucleus and cytoplasm using confocal microscopy and cellular fractionation (Fig. 5c, d). As shown in Fig. 5d, both nuclear and cytosolic stat3 proteins in human cells were decreased by HSP27 downregulation (Fig. 5d, right), whereas most stat3 localized to the cytoplasm in mouse cells expressing HSP25 (Fig. 5d, left). Moreover, stat3 acted as a positive regulator of Daxx expression in both mouse and human cells, suggesting that cellular Daxx is also transcriptionally controlled by stat3 (Fig. 5e). Taken together, our observations suggest two different ways by which cellular Daxx levels in human and mouse cells are controlled; cellular levels of Daxx regulated by HSP27 downregulation were controlled by both proteasomal degradation and stat3-dependent transcriptional induction, whereas in murine cells, the regulation of Daxx levels by HSP25 resulted mainly from stat3-dependent transcription.

### HSP25 structural domain responsible for stat3 mobilization to the nucleus

**Fig. 6:**
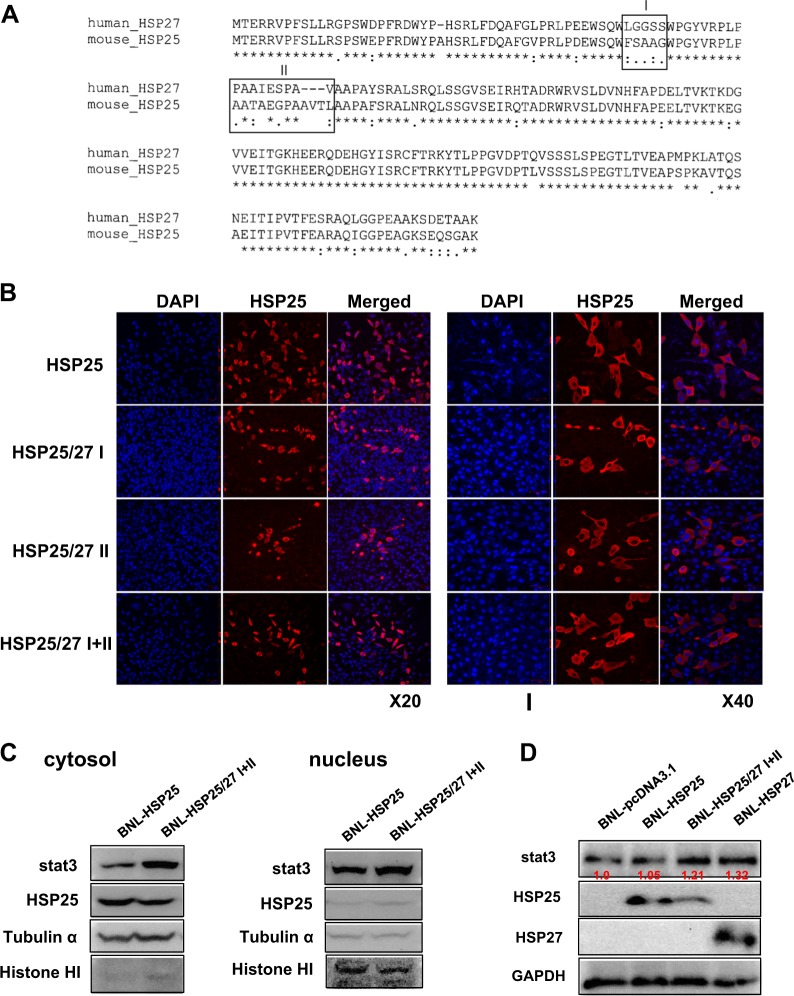
HSP25 structural domain responsible for stat3 mobilization to nucleus. **a** Cellular Daxx levels were examined by the transfection of siRNA against stat3 into A375 and MiaPaCa-2 human cancer cells (left) and BNL and BNL-HSP25 mouse cancer cells (right). **b** Amino acid sequence alignment between human HSP27 and mouse HSP25 to identify the two most variable regions between HSP27 and HSP25 (domains I, II). These regions in HSP27 were replaced with those from HSP25 domain to identify the HSP25 structural domain(s) responsible for impeding stat3 mobilization to the nucleus. **c** The distribution of HSP25 was examined using confocal immunofluorescence. Cellular HSP25 was detected with species-specific primary anti-HSP25 antibody conjugated to Alexa Fluor 594. Changes in the cytoplasmic/nuclear localization of HSP25 were examined in the BNL mouse cell line after its transfection with pcDNA3.1-HSP25 as a control or pcDNA3.1-HSP25 with domains I, or II or both from HSP27. **d** The cytoplasmic/nuclear localization of stat3 was examined in the BNL mouse cell line after its transfection with pcDNA3.1-HSP25 or pcDNA3.1-HSP25 with domains I + II of HSP27 following fractionation into cytoplasmic and nuclear fractions using cytoplasmic (tubulin α) and nuclear (histone H1) marker proteins, respectively. **e** Cellular levels of stat3 were examined after transfection of the BNL cell line with HSP25, HSP25/27 domains I + II, or HSP27. The numbers indicate the band intensities relative to that of BNL-pcDNA3.1 after the band intensities were measured with a densitometer

To identify the domain(s) of HSP25 responsible for the cytoplasmic localization of HSP25 and its inhibition of stat3 nuclear translocation, various chimeric constructs of HSP25 containing domains I, II, or I + II of HSP27 were constructed, and any changes in the localization of HSP25 and stat3 were examined (Fig. 6a). Domains I and II of HSP25 and HSP27 are the most divergent regions between these proteins; therefore, these regions of HSP27 were swapped for the corresponding regions of HSP25. Both domains I and II of HSP27 were responsible for mobilizing HSP25 to the nucleus (Fig. 6b) and mediating the nuclear translocation of stat3 (Fig. 6c).

Unexpectedly, both cytosolic and nucleic levels of stat3 increased (rather than nuclear levels only) after the introduction of HSP25/27 I + II into the BNL mouse cell line. To determine whether HSP25/27 protects STAT3 from proteolytic degradation, cellular levels of stat3 were examined after the introduction of HSP25, HSP25/27 domains I + II, or HSP27; interestingly, increased levels of stat3 were found in BNL cells with HSP25/27 domains I + II or HSP27 (Fig. 6d). This hypothesis originated from the results in Fig. 5a, which shows that the interaction between HSP27 and stat3 was stronger than that between HSP25 and stat3. We found no interaction between stat3 and HSP25/27 domains I + II (Supplementary Fig. 5a); instead, domains I and II appeared to contribute to increasing the expression of stat3 (Supplementary Fig. 5b, c).

### Infection with oncolytic adenovirus expressing Daxx-specific shRNA decreased tumor growth in two different mouse models

**Fig. 7:**
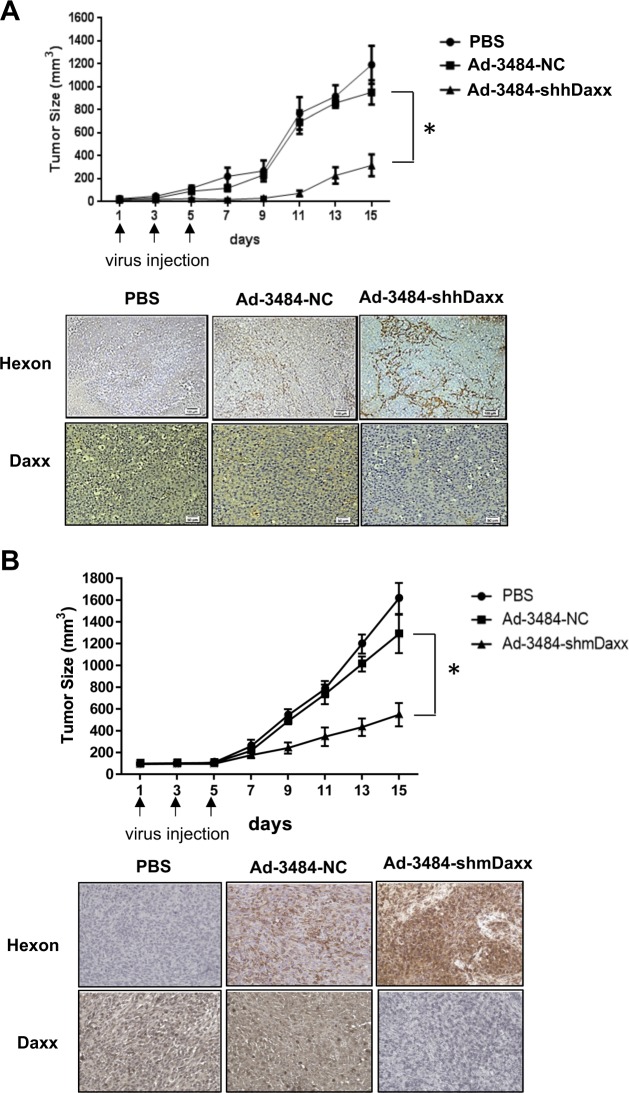
Antitumor effects of oncolytic adenovirus expressing Daxx-specific shRNAs in MiaPaCa-2 or Panc02 tumors in mouse model systems. **a** MiaPaCa-2 cancer cells were established in the abdominal walls of male BALB/c nude mice and injected with Ad-3484-NC as an oncolytic negative control (NC) or Ad-3484-shhDaxx carrying oncolytic shhDaxx. Tumor growth was measured every 2 days for approximately 15 days until necrotic cell death (upper). Error bars represent standard errors from five mice in each experimental group. After established tumors were infected with oncolytic adenovirus expressing human Daxx-specific shRNA or oncolytic adenovirus expressing NC shRNA, tumor tissue sections were prepared for immunohistochemical examination of Daxx expression (lower). **b** Panc02-myrAkt cancer cells were established in the abdominal walls of male C57BL/6 mice and injected with Ad-3484-NC carrying oncolytic NC or Ad-3484-shmDaxx carrying oncolytic shmDaxx. Tumor growth was measured every 2 days for approximately 15 days (upper). Error bars represent standard errors from five mice in each experimental group. After established tumors were injected with oncolytic adenovirus expressing mouse Daxx-specific shRNA or oncolytic adenovirus expressing NC shRNA, tumor tissue sections were prepared for the immunohistochemical examination of Daxx expression (lower; ×100magnification). The asterisk indicates a significant difference between the Ad-3484-NC and Ad-3484-shDaxx groups (*P* < 0.01)

After confirming that Daxx was downregulated following treatment with an oncolytic adenovirus expressing Daxx-specific shRNA in vitro (Supplementary Fig. 4) and that viral production was consequently increased (Fig. 1c), we determined whether an oncolytic adenovirus expressing Daxx-specific shRNA was able to enhance oncolysis in vivo (Fig. 7). For this purpose, we generated BALB/c nude mice harboring MiaPaCa-2 xenografts and C57BL/6 mice harboring Panc02-myrAkt syngeneic tumor grafts. We then examined tumor growth inhibition following the infection of each mouse model with oncolytic adenoviruses expressing Daxx-specific shRNA or negative control (NC; PBS or NC-shRNA-expressing oncolytic adenovirus). As expected, tumor growth inhibition was proportional to Daxx downregulation in both human-derived tumor xenografts (Fig. 7a) and mouse-derived syngeneic tumor grafts (Fig. 7b), suggesting that Daxx downregulation serves as a key strategy to enhance oncolysis in murine tumors.

## Discussion

A previous study indicated that E3 ubiquitin ligases such as E1B55K may be responsible for Daxx degradation^9,30^. However, in our system, Daxx degradation through its ubiquitination after HSP27 downregulation was tenuous instead, ubiquitin-independent proteasomal degradation of Daxx was observed. Here, we provide evidence of distinctly different mechanisms of Daxx regulation at both the transcriptional and posttranslational levels in human and mouse cancer cells. First, we demonstrated that cellular levels of Daxx are the main limiting factor for oncolytic adenoviral replication irrespective of the presence of E1B55K or wild-type p53 in murine cells. We also demonstrated that the induction of HSP25 increases oncolytic adenoviral replication in mouse cancer cells via Daxx downregulation and that Daxx expression is tightly regulated by the actions of both HSP25 and stat3 in mouse cancer cells. HSP25 also likely functions as a p53 suppressor that renders cells capable of efficient viral replication in mice^22^, similar to the effects of viral E1B55K in both human and mouse cells. Here, we found that HSP27 expression was strongly correlated with Daxx expression in human cancer cells and that enhanced viral replication was induced by HSP27 downregulation depending on the cellular p53 status; thus, HSP27 and HSP25 perform distinctly different roles in human and murine cancer cells (Supplementary Fig. 3, Figs. 2d, e, 3d). These differences also arise from the differential localization of these two homologs in the nucleus and cytoplasm. Furthermore, different mechanisms governed Daxx degradation in human and mouse cancer cells: human Daxx degradation induced by HSP27 downregulation was mainly the result of ubiquitin-independent proteasomal degradation; on the other hand, Daxx in BNL cells expressing HSP25 was not degraded by the proteasome, suggesting that mouse Daxx resists proteasomal degradation. Unlike the HSP27-Daxx regulatory system in human cells, HSP25-mediated regulation of Daxx expression in mouse cells appeared to occur via regulation of the nuclear translocation of stat3, a transcription factor that induces Daxx expression. This finding suggests that the HSP25–stat3 interaction controls Daxx expression at mainly the transcriptional level, whereas a more complex mechanism involving both the HSP27-mediated regulation of Daxx proteasomal degradation and the stat3-dependent induction of Daxx transcription controls cellular Daxx levels in human cells.

Taken together, these results suggest that oncolytic adenovirus without E1B55K can replicate well in mouse cells with even wild-type p53 when HSP25 is induced to act as a cellular counterpart to E1B55K. In mouse cells, two main proteins are involved in Daxx-related viral replication: HSP25, which acts as a negative regulator, and stat3, which acts as a positive regulator. During the stationary phase, HSP25 is rarely expressed in cells, allowing stat3 to freely translocate into the nucleus, where it functions as a transcription factor to induce Daxx expression and consequently decrease adenoviral replication. Upon HSP25 induction, HSP25 sequesters stat3 and prevents it from moving into the nucleus, resulting in a decrease in Daxx expression and a consequent increase in adenoviral production. Regulation by the combination of HSP25/stat3 is fairly simple in mouse cells, with HSP25 acting as a negative regulator of Daxx and stat3 acting as a positive regulator of Daxx.

**Fig. 8:**
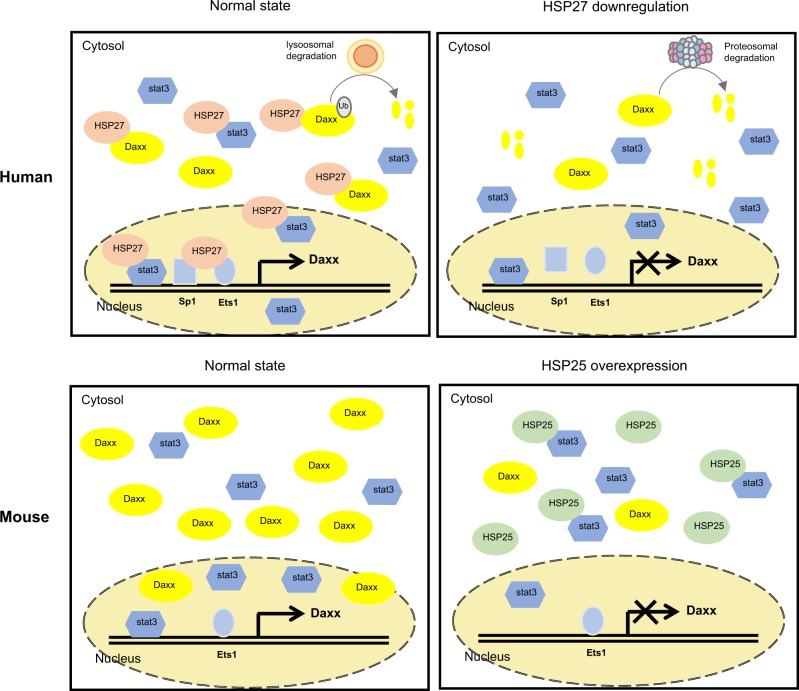
Schematic diagram of the molecular mechanism by which cellular Daxx is differentially regulated in humans and mice. Mouse Daxx is primarily regulated at the transcriptional level via HSP25-induced inhibition of stat3 translocation to the nucleus. In contrast, human HSP27 positively affects stat3 activity to increase human Daxx transcriptional expression. In addition, human Daxx, but not mouse Daxx, is degraded by ubiquitin-dependent lysosomal degradation, while HSP27 downregulation-induced Daxx degradation proceeds through ubiquitin-independent proteasomal degradation

In conclusion, our study reveals that high levels of Daxx due to the decreased expression of HSP25 in mouse cells result in inefficient adenoviral replication. Furthermore, Daxx downregulation is likely to play a crucial role in adenoviral replication; thus, Daxx downregulation may proffer a viable approach to enhancing oncolysis by oncolytic adenoviruses both with and without E1B55K, which extends its usefulness. Figure 8 is a comparative schematic diagram showing cellular Daxx regulation in human and mouse cells. A better understanding of the molecular mechanisms underlying the regulation of Daxx by human HSP27 or mouse HSP25 contributes to the field of viral gene therapy by filling a vital knowledge gap on differential adenoviral replication in cells of human or mouse origin.

## Supplementary information

supple fig 1

supple fig 2

supple fig 3

supple fig 4

supple fig 5

supple fig 6

supple fig 7

supple fig 8
