# Supplementary figures and images for: High levels of Daxx due to low cellular levels of HSP25 in murine cancer cells result in inefficient adenovirus replication
Source: Exp Mol Med. 2019 Oct 15;51(10):122. doi: 10.1038/s12276-019-0321-4 (PMC6802665; doi:10.1038/s12276-019-0321-4)

## Slide 1
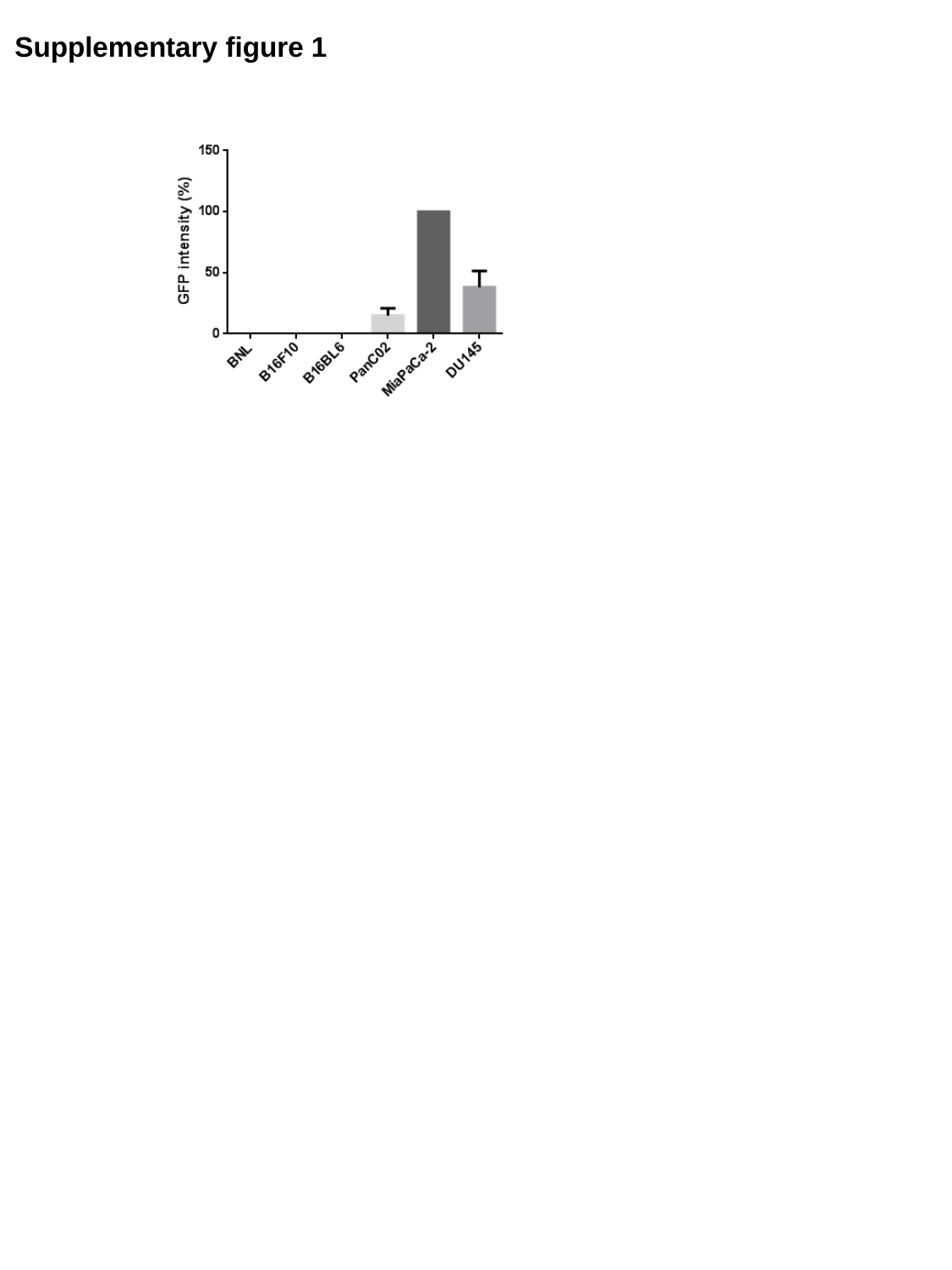

Supplementary figure 1

Supplement: Supplementary file 1 — supple fig 1 [file 12276_2019_321_MOESM1_ESM.pptx]

## Slide 1
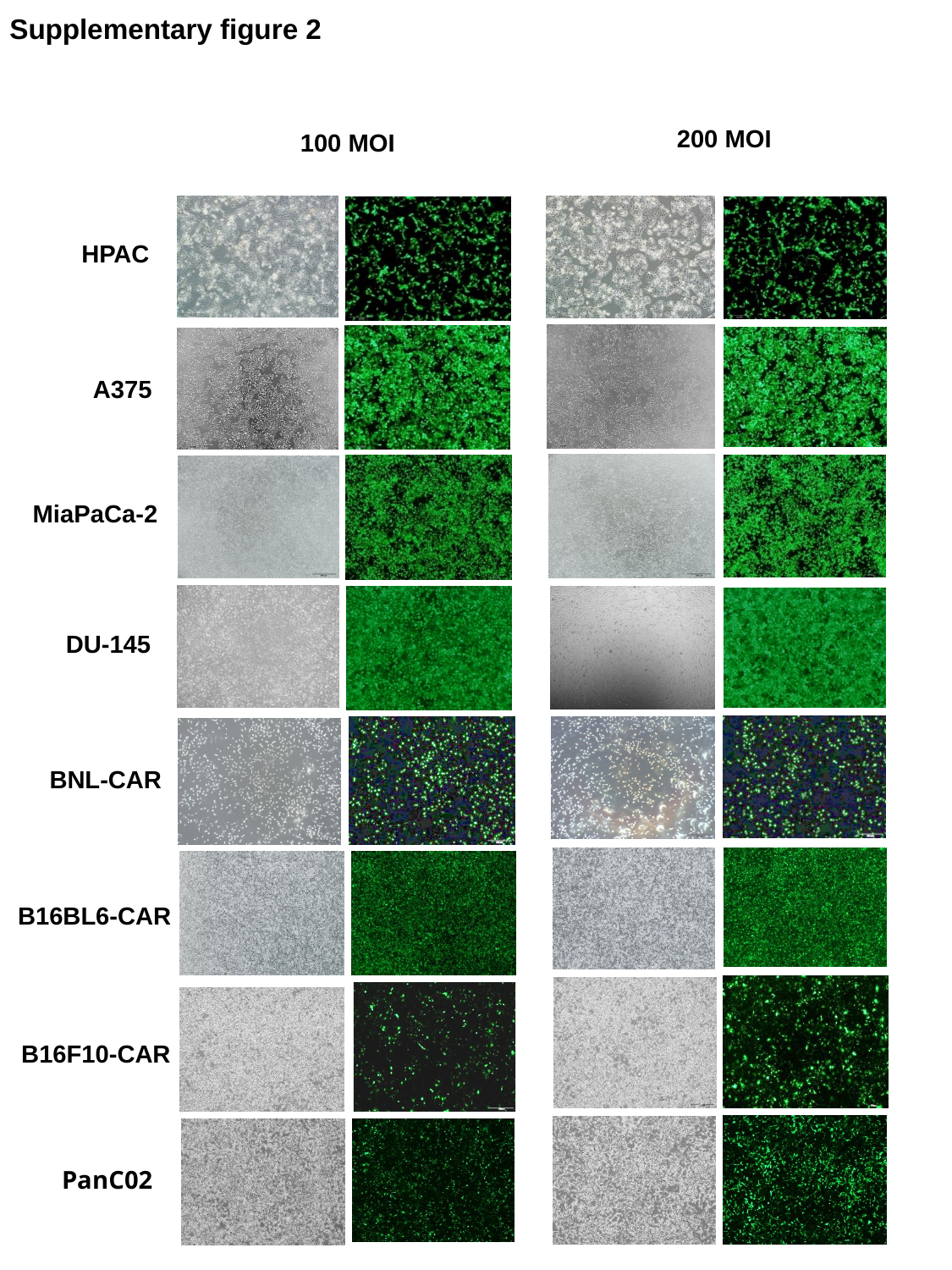

Supplementary figure 2
200 MOI
100 MOI
HPAC
A375
MiaPaCa-2
DU-145
BNL-CAR
B16BL6-CAR
B16F10-CAR
PanC02

Supplement: Supplementary file 2 — supple fig 2 [file 12276_2019_321_MOESM2_ESM.pptx]

## Slide 1
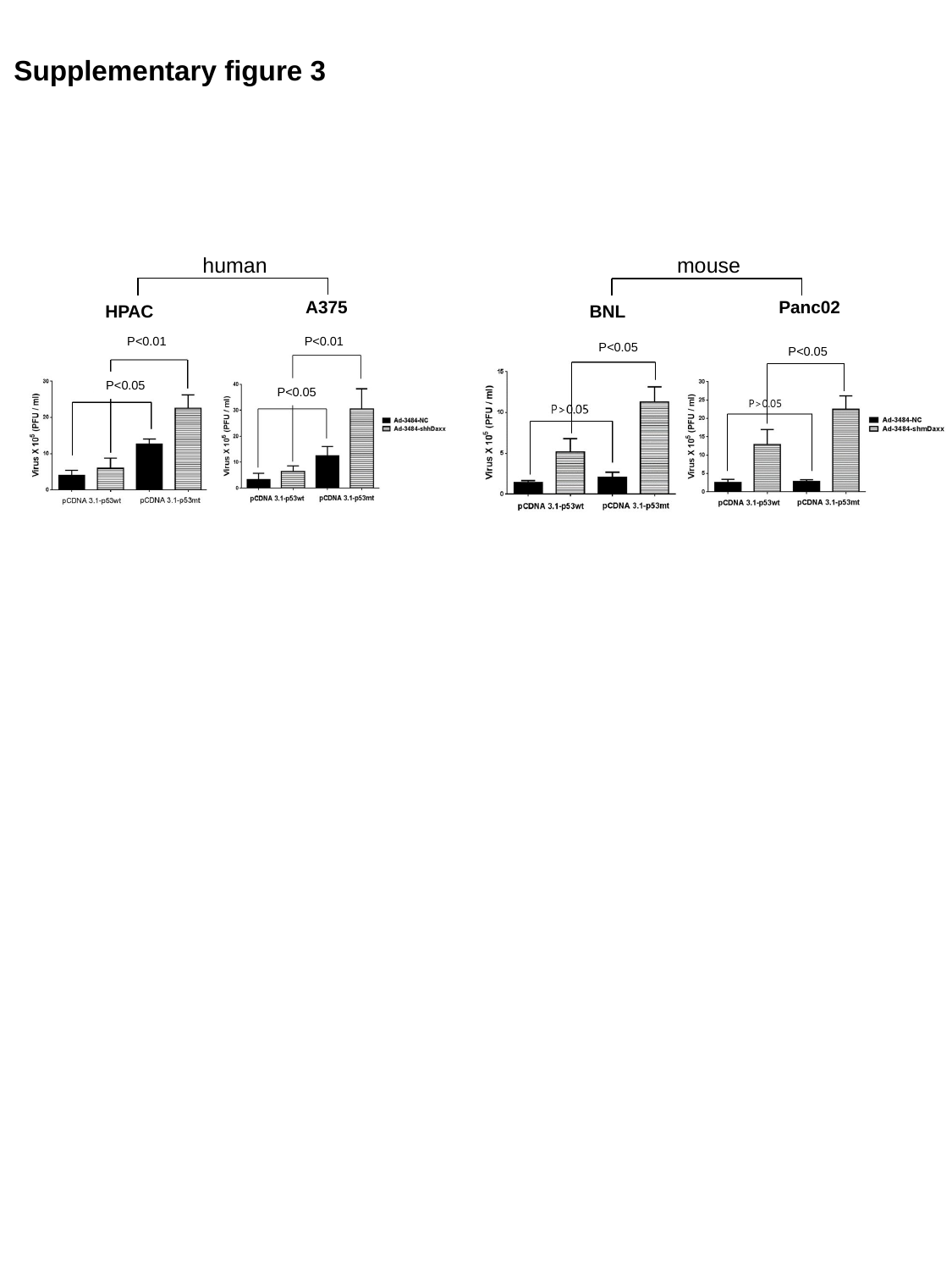

Supplementary figure 3
human
mouse
A375
Panc02
HPAC
BNL
P<0.01
P<0.01
P<0.05
P<0.05
P<0.05
P<0.05

Supplement: Supplementary file 3 — supple fig 3 [file 12276_2019_321_MOESM3_ESM.pptx]

## Slide 1
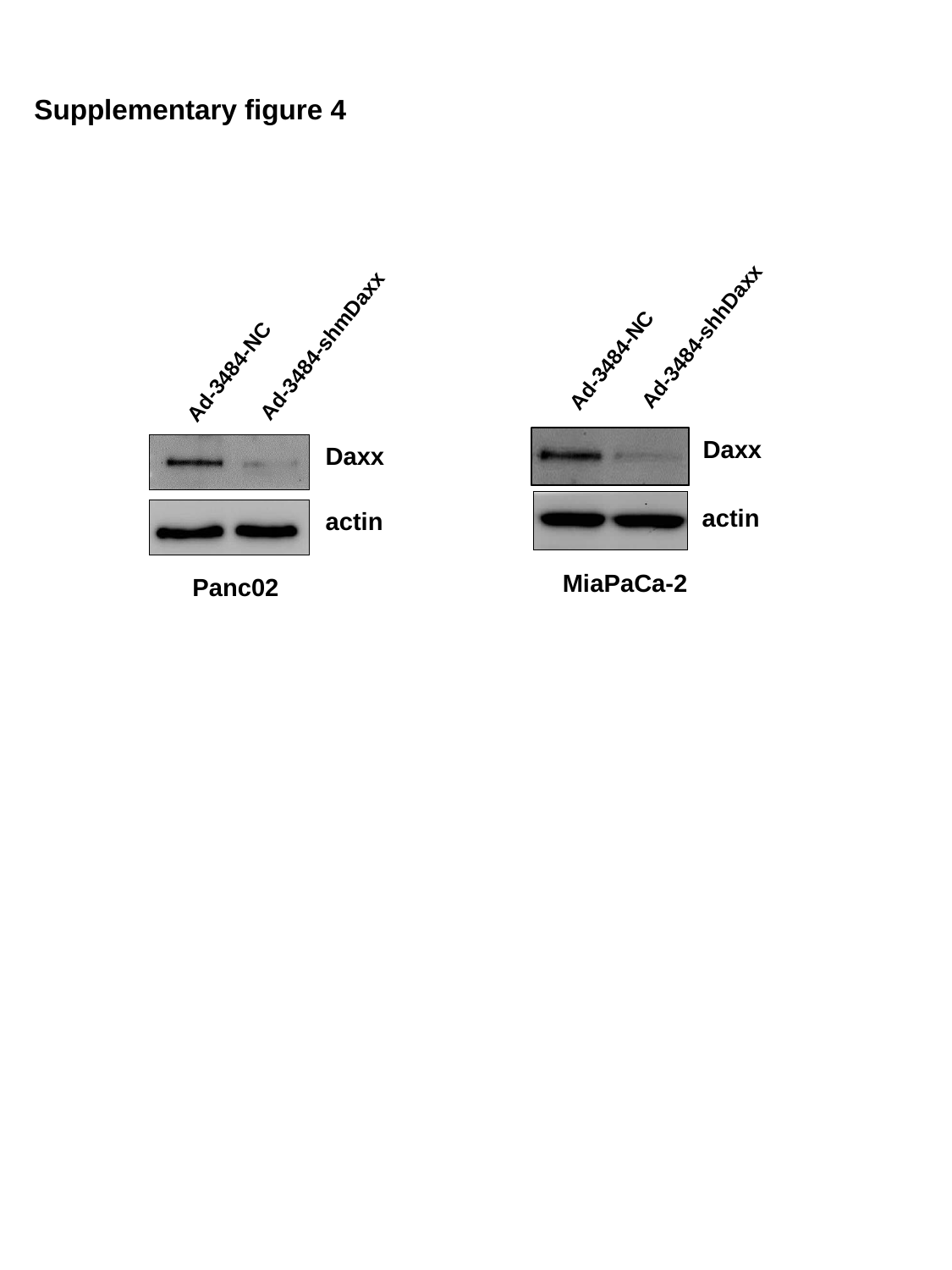

Supplementary figure 4
Ad-3484-shhDaxx
Ad-3484-shmDaxx
Ad-3484-NC
Ad-3484-NC
Daxx
Daxx
actin
actin
MiaPaCa-2
Panc02

Supplement: Supplementary file 4 — supple fig 4 [file 12276_2019_321_MOESM4_ESM.pptx]
