# Supplementary material for: High levels of Daxx due to low cellular levels of HSP25 in murine cancer cells result in inefficient adenovirus replication
Source: Exp Mol Med. 2019 Oct 15;51(10):122. doi: 10.1038/s12276-019-0321-4 (PMC6802665; doi:10.1038/s12276-019-0321-4)
Supplement: Supplementary file 5 — supple fig 5 [file 12276_2019_321_MOESM5_ESM.pptx]

## Slide 1
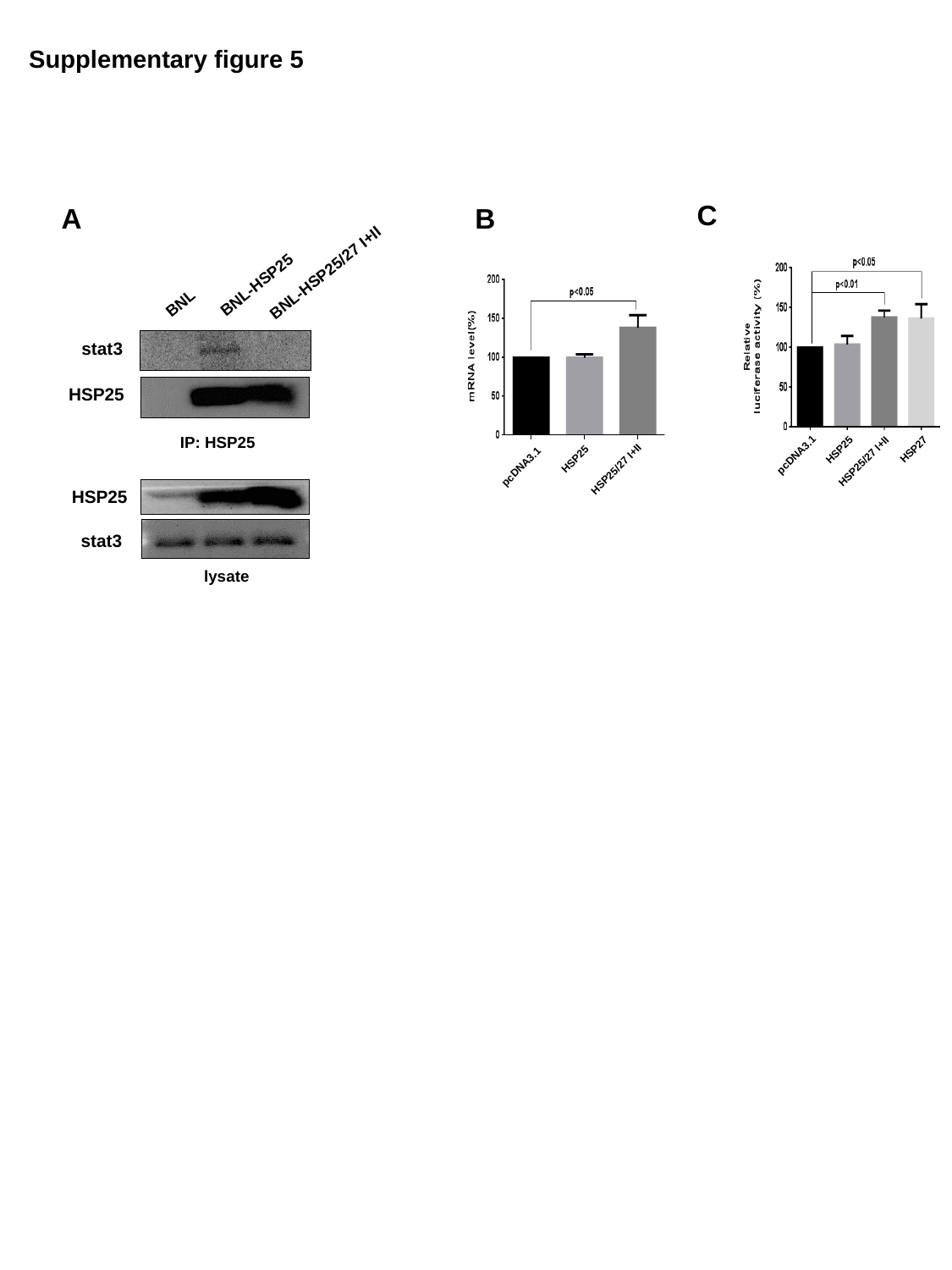

Supplementary figure 5
C
A
B
HSP27
HSP25
pcDNA3.1
HSP25/27 I+II
BNL-HSP25/27 I+II
BNL-HSP25
HSP25
pcDNA3.1
HSP25/27 I+II
BNL
stat3
HSP25
IP: HSP25
HSP25
stat3
lysate
