# Supplementary material for: High levels of Daxx due to low cellular levels of HSP25 in murine cancer cells result in inefficient adenovirus replication
Source: Exp Mol Med. 2019 Oct 15;51(10):122. doi: 10.1038/s12276-019-0321-4 (PMC6802665; doi:10.1038/s12276-019-0321-4)
Supplement: Supplementary file 6 — supple fig 6 [file 12276_2019_321_MOESM6_ESM.pptx]

## Slide 1
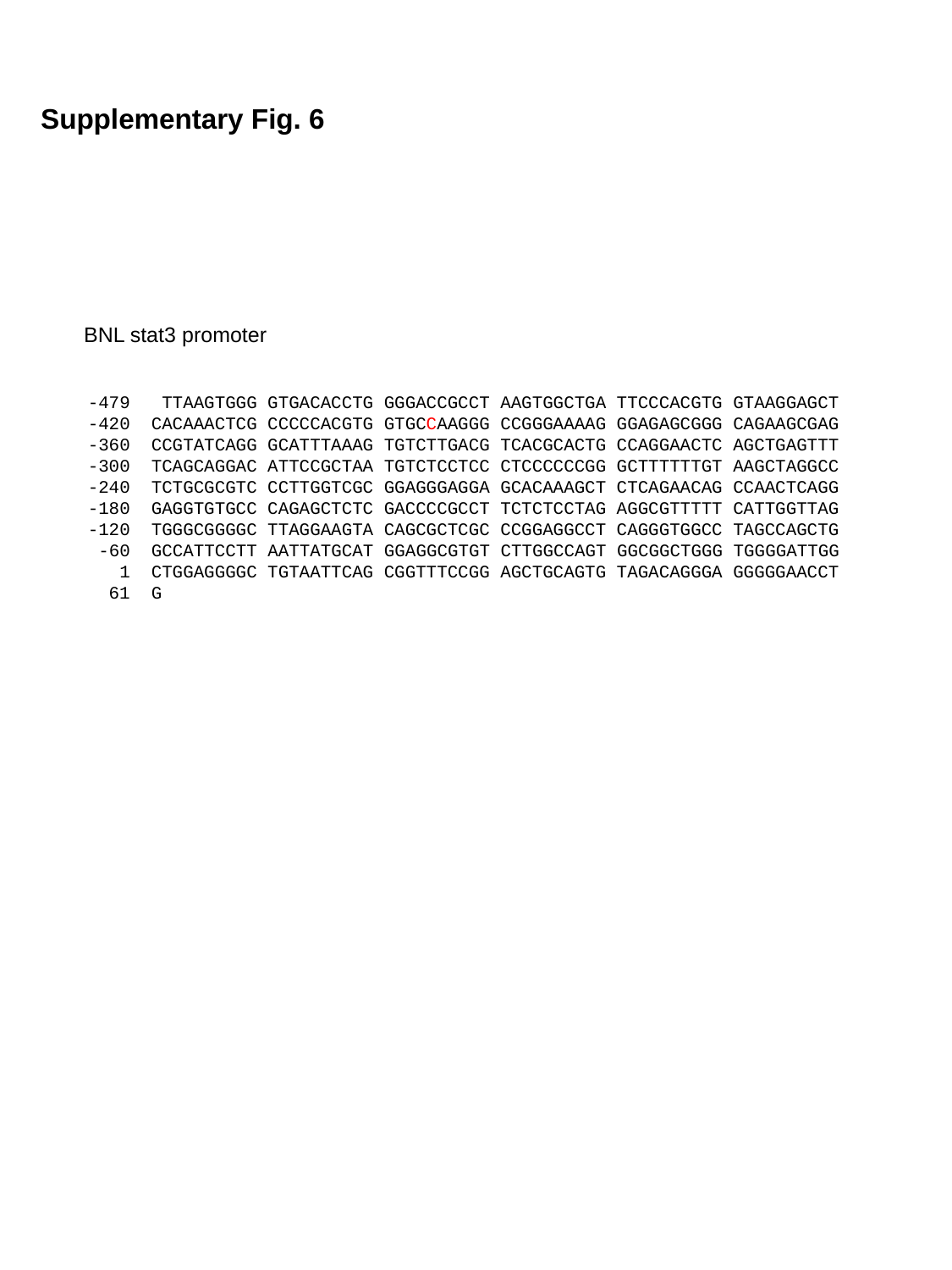

Supplementary Fig. 6
BNL stat3 promoter
-479 TTAAGTGGG GTGACACCTG GGGACCGCCT AAGTGGCTGA TTCCCACGTG GTAAGGAGCT
-420 CACAAACTCG CCCCCACGTG GTGCCAAGGG CCGGGAAAAG GGAGAGCGGG CAGAAGCGAG
-360 CCGTATCAGG GCATTTAAAG TGTCTTGACG TCACGCACTG CCAGGAACTC AGCTGAGTTT
-300 TCAGCAGGAC ATTCCGCTAA TGTCTCCTCC CTCCCCCCGG GCTTTTTTGT AAGCTAGGCC
-240 TCTGCGCGTC CCTTGGTCGC GGAGGGAGGA GCACAAAGCT CTCAGAACAG CCAACTCAGG
-180 GAGGTGTGCC CAGAGCTCTC GACCCCGCCT TCTCTCCTAG AGGCGTTTTT CATTGGTTAG
-120 TGGGCGGGGC TTAGGAAGTA CAGCGCTCGC CCGGAGGCCT CAGGGTGGCC TAGCCAGCTG
 -60 GCCATTCCTT AATTATGCAT GGAGGCGTGT CTTGGCCAGT GGCGGCTGGG TGGGGATTGG
 1 CTGGAGGGGC TGTAATTCAG CGGTTTCCGG AGCTGCAGTG TAGACAGGGA GGGGGAACCT
 61 G
