# Supplementary material for: High levels of Daxx due to low cellular levels of HSP25 in murine cancer cells result in inefficient adenovirus replication
Source: Exp Mol Med. 2019 Oct 15;51(10):122. doi: 10.1038/s12276-019-0321-4 (PMC6802665; doi:10.1038/s12276-019-0321-4)
Supplement: Supplementary file 7 — supple fig 7 [file 12276_2019_321_MOESM7_ESM.pptx]

## Slide 1
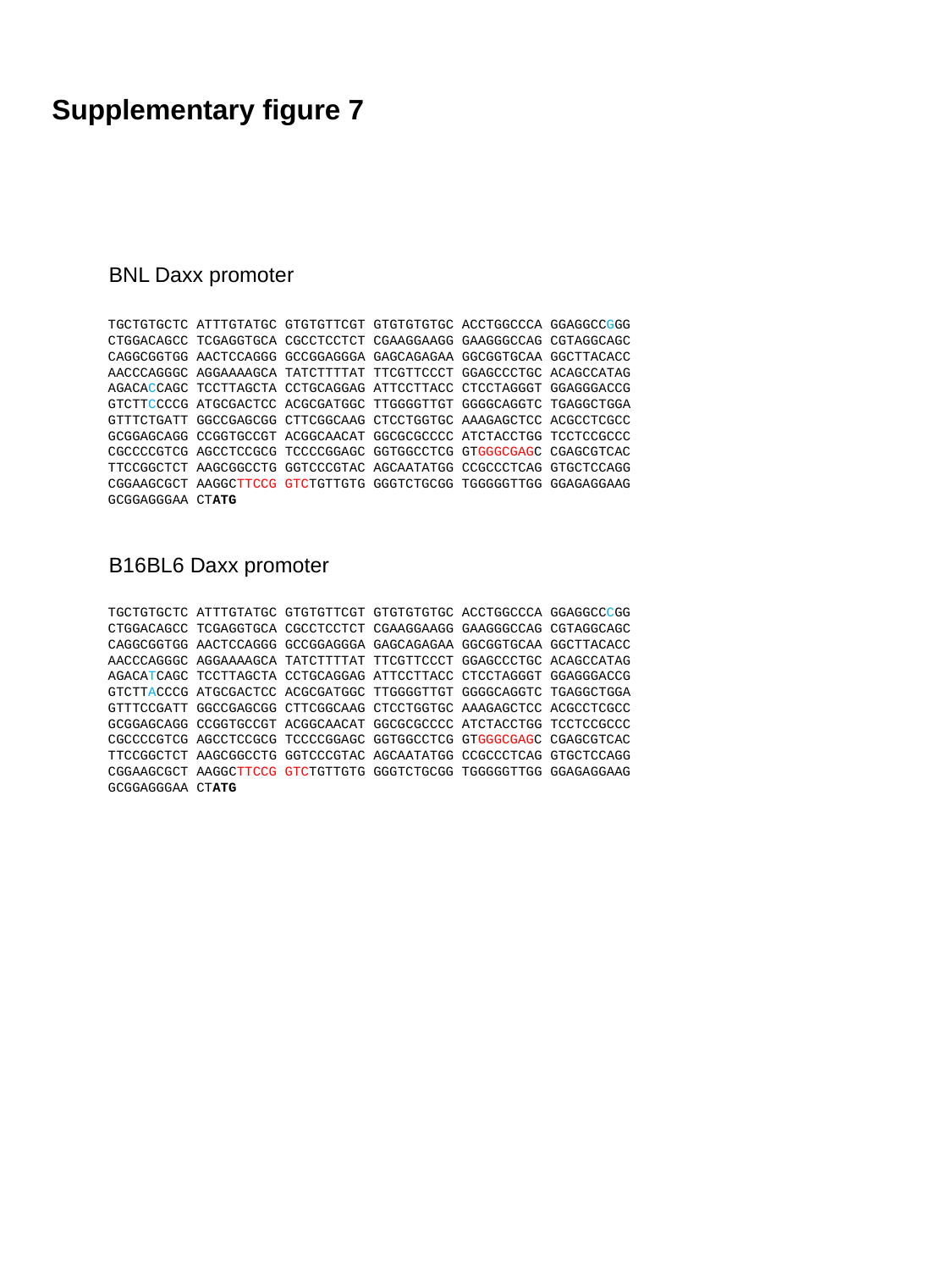

Supplementary figure 7
BNL Daxx promoter
TGCTGTGCTC ATTTGTATGC GTGTGTTCGT GTGTGTGTGC ACCTGGCCCA GGAGGCCGGG CTGGACAGCC TCGAGGTGCA CGCCTCCTCT CGAAGGAAGG GAAGGGCCAG CGTAGGCAGC CAGGCGGTGG AACTCCAGGG GCCGGAGGGA GAGCAGAGAA GGCGGTGCAA GGCTTACACC AACCCAGGGC AGGAAAAGCA TATCTTTTAT TTCGTTCCCT GGAGCCCTGC ACAGCCATAG AGACACCAGC TCCTTAGCTA CCTGCAGGAG ATTCCTTACC CTCCTAGGGT GGAGGGACCG GTCTTCCCCG ATGCGACTCC ACGCGATGGC TTGGGGTTGT GGGGCAGGTC TGAGGCTGGA GTTTCTGATT GGCCGAGCGG CTTCGGCAAG CTCCTGGTGC AAAGAGCTCC ACGCCTCGCC GCGGAGCAGG CCGGTGCCGT ACGGCAACAT GGCGCGCCCC ATCTACCTGG TCCTCCGCCC CGCCCCGTCG AGCCTCCGCG TCCCCGGAGC GGTGGCCTCG GTGGGCGAGC CGAGCGTCAC TTCCGGCTCT AAGCGGCCTG GGTCCCGTAC AGCAATATGG CCGCCCTCAG GTGCTCCAGG CGGAAGCGCT AAGGCTTCCG GTCTGTTGTG GGGTCTGCGG TGGGGGTTGG GGAGAGGAAG GCGGAGGGAA CTATG
B16BL6 Daxx promoter
TGCTGTGCTC ATTTGTATGC GTGTGTTCGT GTGTGTGTGC ACCTGGCCCA GGAGGCCCGG CTGGACAGCC TCGAGGTGCA CGCCTCCTCT CGAAGGAAGG GAAGGGCCAG CGTAGGCAGC CAGGCGGTGG AACTCCAGGG GCCGGAGGGA GAGCAGAGAA GGCGGTGCAA GGCTTACACC AACCCAGGGC AGGAAAAGCA TATCTTTTAT TTCGTTCCCT GGAGCCCTGC ACAGCCATAG AGACATCAGC TCCTTAGCTA CCTGCAGGAG ATTCCTTACC CTCCTAGGGT GGAGGGACCG GTCTTACCCG ATGCGACTCC ACGCGATGGC TTGGGGTTGT GGGGCAGGTC TGAGGCTGGA GTTTCCGATT GGCCGAGCGG CTTCGGCAAG CTCCTGGTGC AAAGAGCTCC ACGCCTCGCC GCGGAGCAGG CCGGTGCCGT ACGGCAACAT GGCGCGCCCC ATCTACCTGG TCCTCCGCCC CGCCCCGTCG AGCCTCCGCG TCCCCGGAGC GGTGGCCTCG GTGGGCGAGC CGAGCGTCAC TTCCGGCTCT AAGCGGCCTG GGTCCCGTAC AGCAATATGG CCGCCCTCAG GTGCTCCAGG CGGAAGCGCT AAGGCTTCCG GTCTGTTGTG GGGTCTGCGG TGGGGGTTGG GGAGAGGAAG GCGGAGGGAA CTATG
