# Supplementary material for: High levels of Daxx due to low cellular levels of HSP25 in murine cancer cells result in inefficient adenovirus replication
Source: Exp Mol Med. 2019 Oct 15;51(10):122. doi: 10.1038/s12276-019-0321-4 (PMC6802665; doi:10.1038/s12276-019-0321-4)
Supplement: Supplementary file 8 — supple fig 8 [file 12276_2019_321_MOESM8_ESM.pptx]

## Slide 1
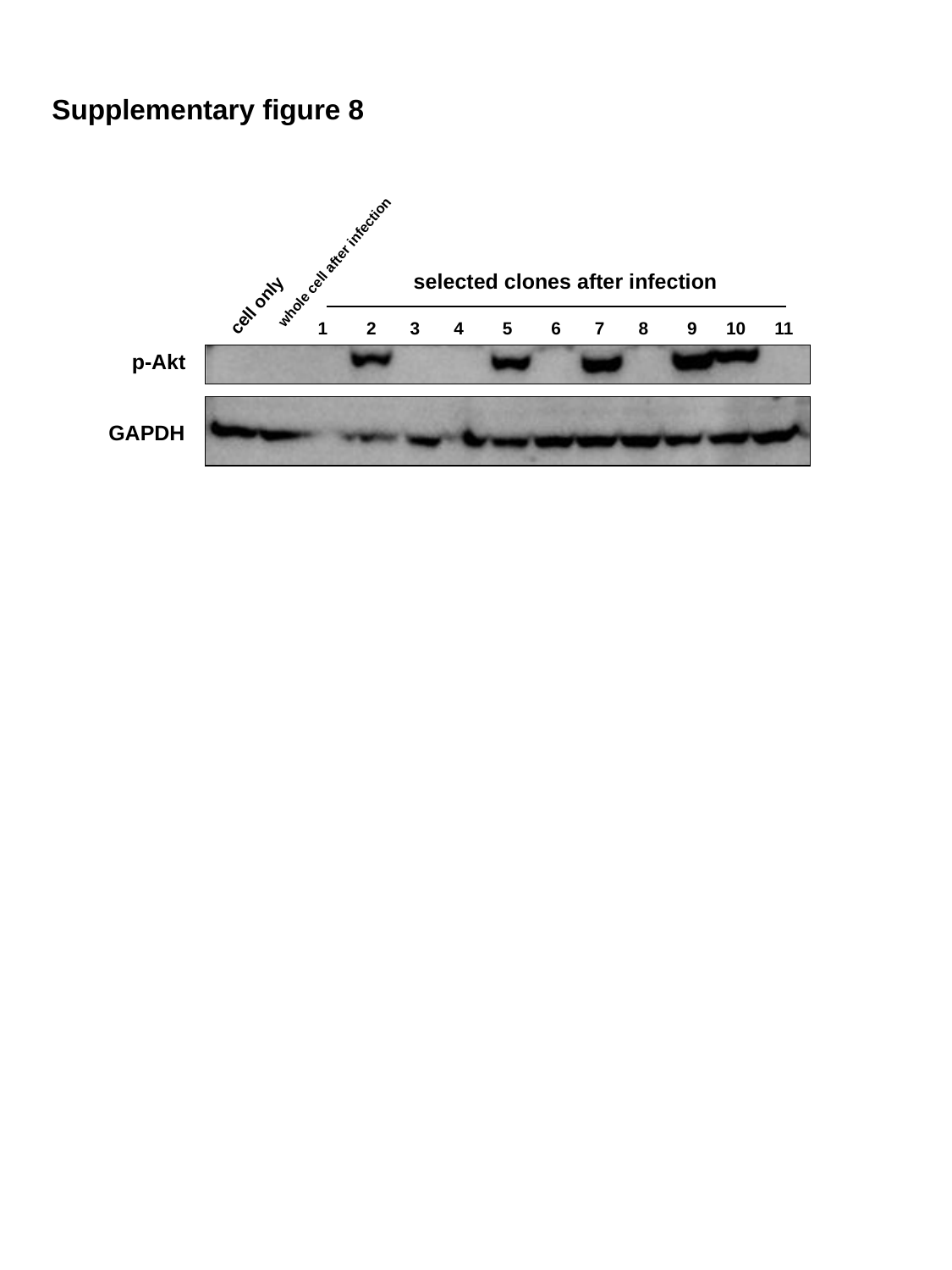

Supplementary figure 8
whole cell after infection
selected clones after infection
cell only
1 2 3 4 5 6 7 8 9 10 11
p-Akt
GAPDH
